# XAF1 drives apoptotic switch of endoplasmic reticulum stress response through destabilization of GRP78 and CHIP

**DOI:** 10.1038/s41419-022-05112-0

**Published:** 2022-07-28

**Authors:** Kyung-Woo Lee, Hui-Ra Hong, Ji-Sun Lim, Kyung-Phil Ko, Min-Goo Lee, Sung-Gil Chi

**Affiliations:** Department of Life Sciences, Korea University, Seoul, 02841 Republic of Korea

**Keywords:** Tumour-suppressor proteins, Apoptosis, Stress signalling

## Abstract

X-linked inhibitor of apoptosis-associated factor-1 (XAF1) is a stress-inducible tumor suppressor that is commonly inactivated in many human cancers. Despite accumulating evidence for the pro-apoptotic role for XAF1 under various stressful conditions, its involvement in endoplasmic reticulum (ER) stress response remains undefined. Here, we report that XAF1 increases cell sensitivity to ER stress and acts as a molecular switch in unfolded protein response (UPR)-mediated cell-fate decisions favoring apoptosis over adaptive autophagy. Mechanistically, XAF1 interacts with and destabilizes ER stress sensor GRP78 through the assembly of zinc finger protein 313 (ZNF313)-mediated destruction complex. Moreover, XAF1 expression is activated through PERK-Nrf2 signaling and destabilizes C-terminus of Hsc70-interacting protein (CHIP) ubiquitin E3 ligase, thereby blocking CHIP-mediated K63-linked ubiquitination and subsequent phosphorylation of inositol-required enzyme-1α (IRE1α) that is involved in in the adaptive ER stress response. In tumor xenograft assays, *XAF1*^*−/−*^ tumors display substantially lower regression compared to *XAF1*^*+/+*^ tumors in response to cytotoxic dose of ER stress inducer. XAF1 and GRP78 expression show an inverse correlation in human cancer cell lines and primary breast carcinomas. Collectively this study uncovers an important role for XAF1 as a linchpin to govern the sensitivity to ER stress and the outcomes of UPR signaling, illuminating the mechanistic consequence of *XAF1* inactivation in tumorigenesis.

## Introduction

Endoplasmic reticulum (ER) is a central organelle responsible for the synthesis, maturation, and transportation of proteins [1, 2]. Perturbation of ER homeostasis causes the accumulation of excessive unfolded or misfolded proteins, which is referred to as ER stress [3]. ER stress is sensed by a quality control mechanism termed unfolded protein response (UPR), which aims to maintain the proteostasis in the ER lumen by increasing protein folding capacity and misfolded protein degradation [4, 5].

Glucose-regulated protein (GRP78), also called BiP or HSPA5, is a major ER chaperone protein that acts as an ER stress sensor [3]. GRP78 is primarily located in the ER but also detected in the cell surface, cytosol, and mitochondria [6]. In unstressed cells, GRP78 binds to three transmembrane UPR transducers, inositol-required enzyme-1 (IRE1), protein kinase RNA-like endoplasmic reticulum kinase (PERK) and activating transcription factor 6 (ATF6) to keep them in an inactive state. In response to ER stress, GRP78 is redirected from these UPR proteins to misfolded proteins and UPR signaling is initiated to facilitate the proteasomal degradation of misfolded proteins [7, 8]. The maintenance of ER integrity and proteostasis is essential for rapidly growing cancer cells [9, 10]. Expression of GRP78 is associated with ER integrity and enhanced cell survival [11, 12]. High expression of GRP78 is observed in multiple human cancers and correlates with malignant progression and tumor resistance to etoposide, cisplatin, temozolomide, and γ-radiation [4, 13–16].

X-linked inhibitor of apoptosis (XIAP)-associated factor 1 (XAF1) is a pro-apoptotic tumor suppressor that is originally found to antagonize the anti-caspase activity of XIAP [17]. Epigenetic inactivation of *XAF1* due to aberrant promoter hypermethylation is observed in a broad range of human cancers and associates with the stage and grade of many tumors [18–21]. The *XAF1* gene encodes 33 kDa protein that contains seven zinc finger (ZF) domains, suggesting its role in the regulation of protein–protein interaction [17]. XAF1 interacts with many proteins, including zinc finger protein 313 (ZNF313) ubiquitin E3 ligase and homeodomain-interacting protein kinase 2 and can promote apoptosis through multiple XIAP-independent mechanisms [22–24].

*XAF1* transcription is activated in response to various genotoxic, oxidative, and cytokine stresses to drive apoptosis induction [20, 21]. In particular, *XAF1* is strongly activated by interferons and sensitizes cells to the pro-apoptotic actions of interferons and tumor necrosis factor-related apoptosis inducing ligand [23–26]. XAF1 also regulates autophagy, tumor angiogenesis, and G2/M checkpoint of the cell cycle [27, 28]. Our studies show that XAF1 is activated by the p53 tumor suppressor and acts as a molecular switch in p53-mediated cell-fate decisions favoring apoptosis over cell-cycle arrest [23]. XAF1 forms a feedback loop with interferon regulatory factor-1 (IRF-1) and evokes its tumor suppression effect in a highly IRF-1-dependent fashion [29]. Recently, we reported that XAF1 is activated by heavy metals and triggers an apoptotic conversion of stress response by binding and destabilizing metallothionein 2A [30].

Despite accumulating evidence for the apoptosis-promoting role for XAF1 under various stressful conditions, its role in ER stress response remains undefined. In the present study, we found that XAF1 enhances cell sensitivity to ER stress through GRP78 destabilization and its induction drives an apoptotic switch of UPR function by blocking IRE1α phosphorylation. Therefore, this study uncovers an important role for XAF1 in UPR-mediated cell-fate decisions.

## Results

### XAF1 expression enhances apoptotic response to ER stress

**Fig. 1:**
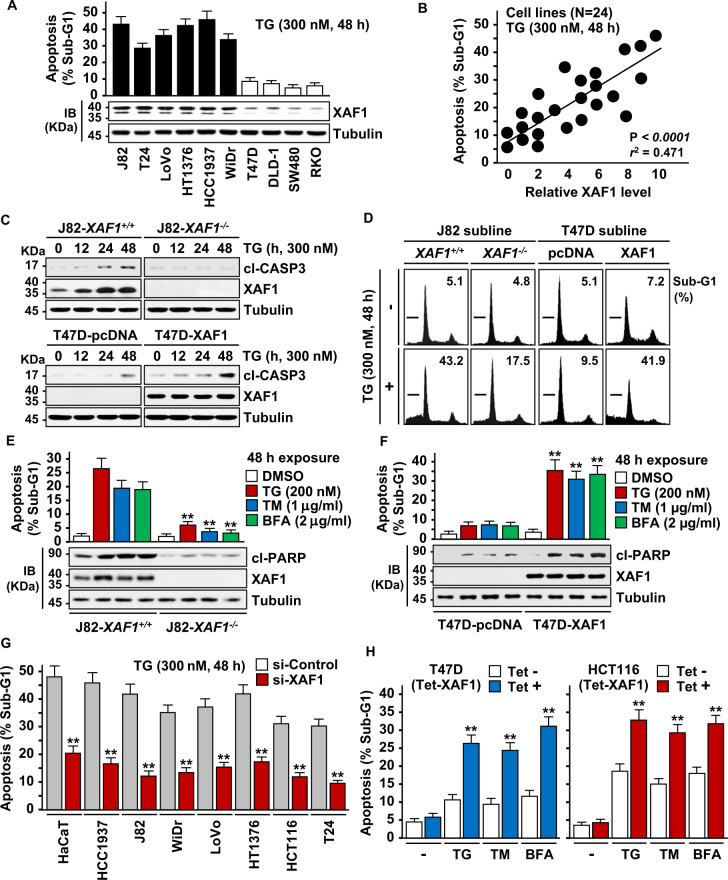
XAF1 expression is associated with cytotoxic ER stress response. **A**, **B** XAF1 expression and its association with apoptotic tumor cell response to TG. Apoptosis was determined by flow cytometric measurement of sub-G1 fraction. IB, immunoblot. *r*^2^, Pearson’s correlation coefficient. **C**–**F** Effect of XAF1 knockout and overexpression on ER stress-induced apoptosis. J82 and T47D sublines were treated with TG, TM, and BFA as indicated. Apoptosis induction was determined by IB assay of cleaved caspase-3 (cl-CASP3) and flow cytometric analysis of sub-G1 fraction. **G** Effect of XAF1 knockdown on TG-induced apoptosis in human cancer cell lines. Cells were transfected with 50 pM of si-Control or si-XAF1 and then exposed to TG. **H** Effect of XAF1 overexpression on ER stress-induced apoptosis. XAF1 induction was achieved by Tet-inducible XAF1 (Tet-XAF1) system. Cells were treated with TG (300 nM), TM (1 μg/ml), or BFA (2 μg/ml) for 48 h. Data represent the mean ± SD of triplicate assays. **P < 0.01 (Student *t*-test).

To explore the role for XAF1 in ER stress response, we initially examined whether it affects apoptotic response to ER stress using 24 human cancer cell lines. A flow cytometric analysis of sub-G1 fraction revealed that XAF1 expression is associated with apoptotic sensitivity to the ER stress inducer thapsigargin (TG) (Fig. 1A, B). Based on this, we tested effect of gene knockout and stable overexpression using J82 (high XAF1) and T47D (low XAF1) cells, respectively. Compared to J82-*XAF1*^*+/+*^, J82-*XAF1*^*−/*^^−^ subline cells exhibited markedly attenuated apoptotic response to TG (43.2% versus 17.5%) and other ER stress inducers, such as tunicamycin (TM) and brefeldin A (BFA) (Fig. 1C–E). It was also recognized that XAF1 expression is strongly induced by the stress inducers in *XAF1*^*+/+*^ cells while *XAF1*^*−/−*^ cells recover the apoptotic sensitivity to TG when XAF1 is restored (Fig. 1C, E and Supplementary Fig. 1A). Consistently, compared to T47D-pcDNA, T47D-XAF1 subline exhibited enhanced apoptotic response to TG (9.5% versus 41.9%) and other stress inducers (Fig. 1D, F). A series of assays using transient knockdown and overexpression or tetracycline-inducible XAF1 (Tet-XAF1) system confirmed that XAF1 increases tumor cell sensitivity to ER stress-mediated apoptosis (Fig. 1G, H and Supplementary Fig. 1B, C).

Autophagy is a representative adaptive response to ER stress [31]. However, autophagy also can lead to cell death, indicating that autophagy pathway can be subverted from a survival to a death function [32]. We asked whether XAF1 promotes apoptosis through the regulation of autophagy. Compared to J82-*XAF1*^*−/−*^ and T47D-pcDNA sublines, J82-*XAF1*^*+/+*^ and T47D-XAF1 sublines displayed higher autophagic response (increased LC3-I/II) (Supplementary Fig. 1D–F). As predicted, blockade of autophagy by 3-MA treatment caused apoptosis reduction in J82-*XAF1*^*+/+*^ and T47D-XAF1 cells. By contrast, in J82-*XAF1*^*−/*^^−^ and T47D-pcDNA cells, 3-MA treatment resulted in apoptosis induction. Immunoblot (IB) assays of autophagy markers, such as Beclin-1, and Atg5-Atg12, in LoVo sublines (*XAF1*^*+/+*^ and *XAF1*^*−/−*^) and T47D (Tet-XAF1) cells also showed that XAF1 induction of apoptosis is associated with its stimulation of autophagy (Supplementary Fig. 1G–I). Consistently, immunofluorescence (IF) assay of LC3B puncta revealed that XAF1 increases TG-induced LC3B puncta and this effect is abrogated by 3-MA (Supplementary Fig. 1J, K). This observation supports that autophagy exerts protective role in XAF1-nonexpressing cells and that XAF1 expression facilitates autophagy to drive ER stress-mediated apoptosis. To define whether XAF1 induction of LC3B puncta results from autophagy activation or its lysosomal accumulation due to inhibition of autophagic flux, effects of 3-MA (inhibitor of early autophagy) and BafA1 (inhibitor of late autophagy) were compared. XAF1 induction of LC3B puncta was blocked by 3-MA but increased by BafA1, indicating that XAF1 facilitates autophagic flux (Supplementary Fig. 1J, K). Together, these support that XAF1 stimulates autophagy-mediated apoptosis under ER stress conditions.

### XAF1 activates ER stress-mediated UPR signaling through GRP78 destabilization

**Fig. 2:**
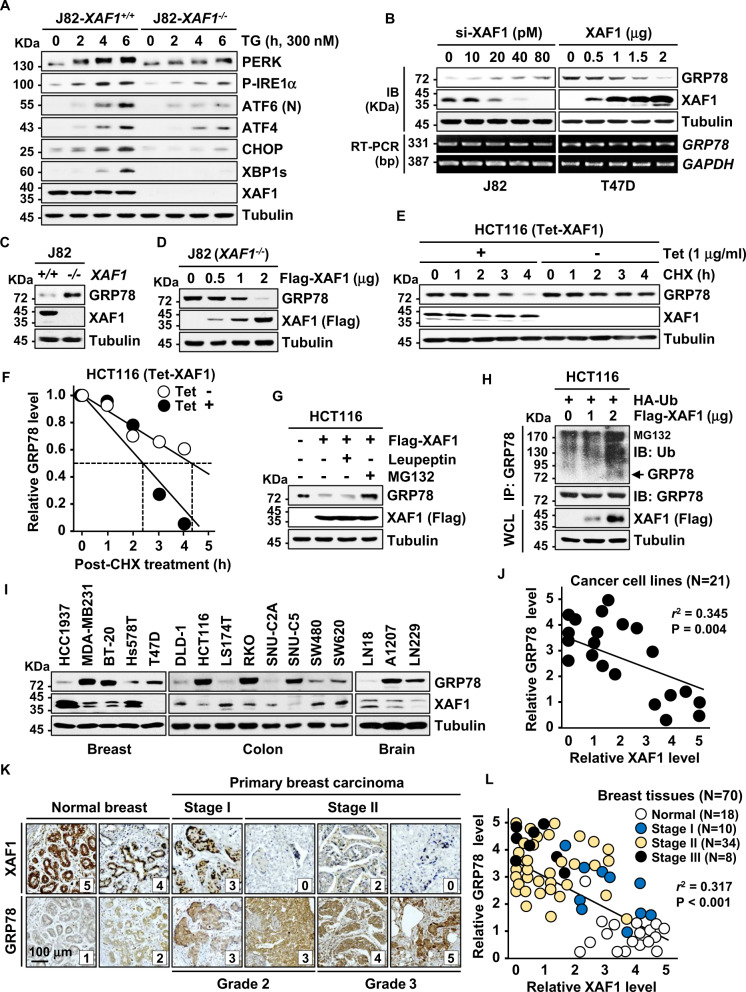
XAF1 induces GRP78 degradation to activate UPR signaling. **A** Comparison of *XAF1*^*+/+*^ and *XAF1*^*−/−*^ sublines of J82 in TG-induced UPR signaling. **B** Effect of XAF1 depletion and expression on GRP78 expression. Cells were transfected with increasing doses of si-XAF1 or XAF1. GRP78 protein and mRNA levels were examined at 48 h after transfection. **C** Comparison of GRP78 protein level in J82-*XAF1*^*+/+*^ and J82-*XAF1*^−/−^ sublines. **D** Effect of XAF1 restoration on GRP78 expression in J82-*XAF1*^−/−^ cells. **E**, **F** A CHX chase experiment showing GRP78-destabilizing effect of XAF1. Cells were exposed to CHX (40 μM) for indicated times. XAF1 expression was achieved using Tet-inducible XAF1 system (Tet-XAF1). **G** Impairment of XAF1-mediated GRP78 reduction by MG132. Cells transfected with Flag-XAF1 were exposed to MG132 (10 μM) or Leupeptin (10 μM) for 6 h. **H** IP assay showing XAF1 induction of GRP78 ubiquitination. HA, hemagglutinin; Ub, ubiquitin; WCL, whole cell lysate. **I**, **J** An inverse correlation of XAF1 and GRP78 protein levels in human cancer cell lines. *r*^2^, Pearson’s correlation coefficient. **K**, **L** IHC assay of XAF1 and GRP78 expression (dark brown) in human breast carcinoma and normal tissues. Relative staining levels were classified as levels 0–5. *r*^2^, Pearson’s correlation coefficient.

Next we examined whether XAF1 affects ER stress-mediated UPR signaling. In response to TG, J82-*XAF1*^*+/+*^ cells displayed faster activation of UPR transducers (PERK, IRE1α, and ATF6) and their effectors (CHOP, XBP1s, elF2α, and ATF4) compared to *XAF1*^*−/−*^ subline cells (Fig. 2A). In T47D cells, UPR activation by TG treatment was facilitated by transient overexpression of XAF1 in a transfection dose-dependent manner while it was delayed by XAF1 depletion in LoVo cells (high XAF1) (Supplementary Fig. 2A, B). Based on this observation, we tested whether XAF1 regulates GRP78, a chaperone protein acting as a master switch of UPR signaling [7, 8]. GRP78 protein but not mRNA expression was markedly increased and decreased by XAF1 depletion and expression, respectively (Fig. 2B and Supplementary Fig. 2C, D). Likewise, a higher GRP78 level was detected in J82-*XAF1*^*−/*^^−^ versus J82-*XAF1*^*+/+*^ cells while it was downregulated by XAF1 restoration (Fig. 2C, D). XAF1 regulates the stability of multiple proteins through interaction with ubiquitin E3 ligases [22, 23, 33]. A cycloheximide (CHX) assay showed that XAF1 induction shortens the half-life of GRP78 protein from approximately 4.3h to 2.4h (Fig. 2E, F). XAF1-mediated GRP78 degradation was completely blocked by the proteasome inhibitor MG132, but not affected by the lysosome inhibitor leupeptin (Fig. 2G). Moreover, immunoprecipitation (IP) assay revealed that XAF1 increases GRP78 ubiquitination, supporting that XAF1 promotes the ubiquitin-mediated proteasomal degradation of GRP78 (Fig. 2H). In addition, a strong inverse correlation was identified between XAF1 and GRP78 levels in 21 human cancer cell lines (Fig. 2I, J). Immunohistochemistry (IHC) study of 70 human breast tissues also identified a frequent reduction of XAF1 in tumor versus normal tissues and its inverse correlation with GRP78 expression (Fig. 2K, L).

### XAF1 promotes GRP78 degradation through direct interaction

**Fig. 3:**
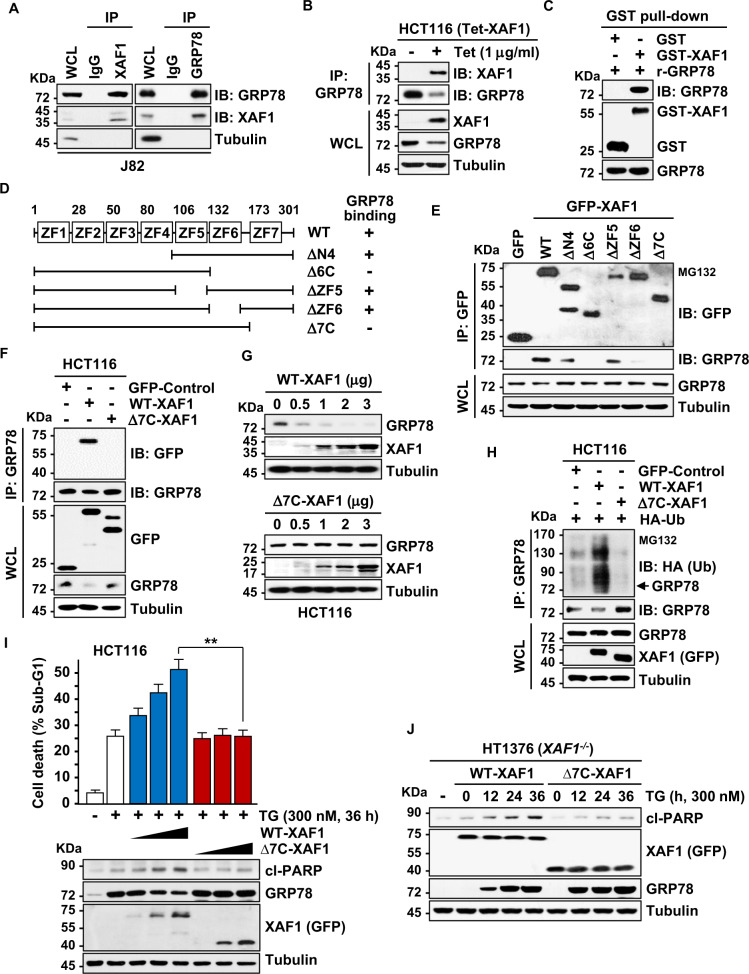
XAF1 binds directly to GRP78. **A**, **B** IP assays showing XAF1 interaction with GRP78. **C** In vitro GST pull-down assay showing the direct interaction of purified GST-XAF1 and recombinant GRP78 proteins. GST, glutathionine S-transferase; r, Recombinant. **D** Deletion mutants of XAF1 and their GRP78-binding status. ZF, zinc finger; WT, wild-type. **E**–**G** IP assays showing a critical role for ZF7-containing C-terminal region (7C) of XAF1 in binding and downregulation of GRP78. **H** No GRP78 ubiquitination-inducing ability of Δ7C-XAF1. **I**, **J** No apoptosis-promoting activity of Δ7C-XAF1. Cells were transfected with either WT-XAF1 or Δ7C-XAF1 and then treated with TG (300 nM). Apoptosis induction was determined by flow cytometric analysis of sub-G1 fraction and IB assay of cleaved PARP level. Data represent the mean ± SD of triplicate assays. **P < 0.01 (Student *t*-test).

To define the mechanism underlying the XAF1 degradation of GRP78, we examined whether XAF1 binds to GRP78. IP and in vitro GST pull-down assays revealed that XAF1 binds to GRP78 (Fig. 3A–C). IF assay of GRP78 and XAF1 cellular localization supported their interaction (Supplementary Fig. 3A). Using a series of deletion mutants, we identified that the C-terminal region including zinc finger 7 (ZF7) domain of XAF1 is responsible for the interaction (Fig. 3D–F). Unlike wild-type (WT) XAF1, a mutant XAF1 lacking the GRP78-interacting region (Δ7C-XAF1) showed no activity to induce ubiquitination and reduction of GRP78 (Fig. 3G, H). As predicted, Δ7C-XAF1 failed to promote TG-induced apoptosis (Fig. 3I, J and Supplementary Fig. 3B). These indicate that XAF1 enhances apoptotic ER stress response through interaction-mediated destabilization of GRP78.

### XAF1 destabilizes GRP78 through the assembly of ZNF313-mediated destruction complex

**Fig. 4:**
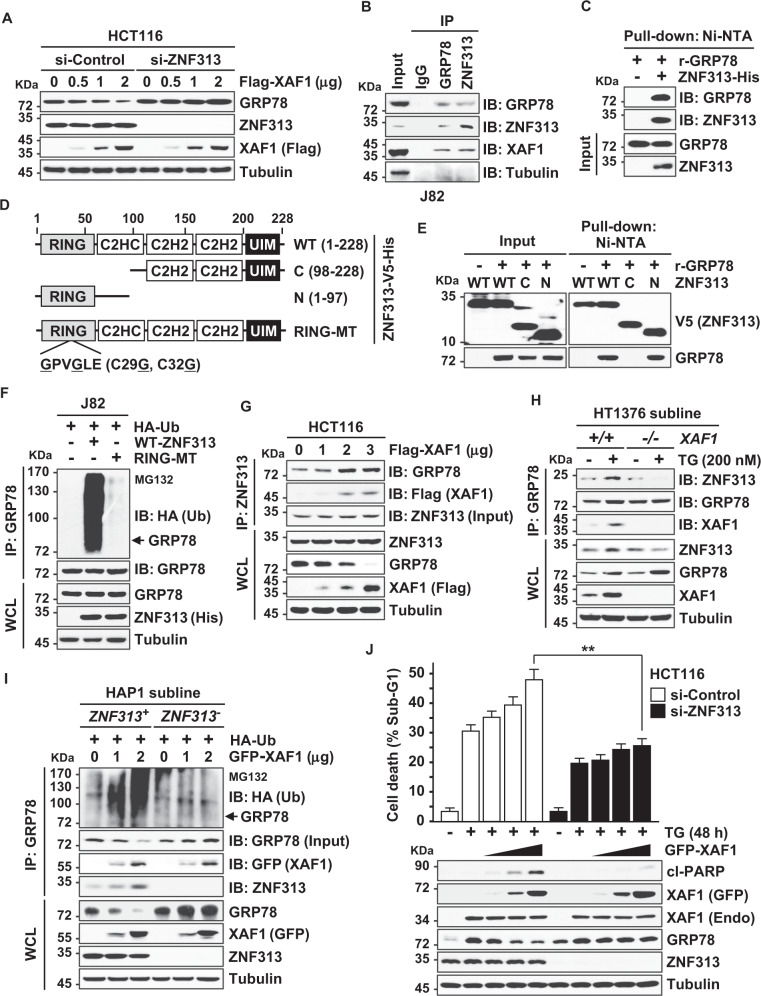
XAF1 promotes ZNF313-GRP78 interaction to facilitate GRP78 ubiquitination. **A** Effect of ZNF313 depletion on XAF1-mediated GRP78 reduction. Cells were transfected with 50 pM of si-Control or si-ZNF313 and IB assay was carried out at 48 h after transfection. **B** IP assay showing XAF1 interaction with GRP78. **C** In vitro GST pull-down assay showing the direct interaction of purified ZNF313-His and recombinant GRP78 (r-GRP78). **D** Construction of deletion mutants of ZNF313 for characterization of GRP78-interacting region. RING, Really Interesting New Gene; UIM, ubiquitin-interacting motif; MT, mutant. **E** A Ni-NTA pull-down assay for the GRP78-interacting activity of WT and deletion mutants of ZNF313. **F** Loss of GRP78-ubiquitinating activity by RING mutation. **G** XAF1 stimulation of ZNF313-GRP78 interaction. IP was performed using equal amount of ZNF313 input. **H** Effect of *XAF1* knockout on TG-induced ZNF313-GRP78 interaction. **I** Validation of a ZNF313 dependency of XAF1 stimulation of ZNF313-GRP78 interaction and GRP78 ubiquitination. XAF1 effect was compared in HAP1 *ZNF313*^*+*^ and *ZNF313*^*-*^ subline cells. **J** Effect of ZNF313 depletion on XAF1 stimulation of TG-mediated apoptosis. Data represent the mean ± SD of triplicate assays. **P < 0.01 (Student *t*-test).

To identify an E3 ligase responsible for XAF1-induced GRP78 ubiquitination, we tested the involvement of ZNF313, which is known to interact with XAF1 [22]. XAF1-induced GRP78 reduction was totally impaired in ZNF313-depleted cells while GRP78 level was markedly decreased by ectopic overexpression of ZNF313 (Fig. 4A and Supplementary Fig. 4A, B). Moreover, ZNF313 overexpression strongly increased ubiquitination of GRP78 (Supplementary Fig. 4C). A series of interaction assays revealed that ZNF313 binds directly to GRP78 through the N-terminus including the RING domain (Fig. 4B, C and Supplementary Fig. 4C, D). Unlike WT-ZNF313, a RING mutant (RING-MT), which has C-to-G sequence replacement at codons 29 and 32 in the RING domain, failed to interact with GRP78 and showed no activity to increase GRP78 ubiquitination (Fig. 4D–F). Furthermore, IP assay showed that ZNF313-GRP78 interaction is reinforced in a XAF1 transfection dose-associated manner (Fig. 4G). TG treatment increased ZNF313-GRP78 interaction in *XAF1*^*+/+*^ but not in *XAF1*^*−/−*^ subline of HT1376 cells (Fig. 4H). Moreover, XAF1-induced ubiquitination and downregulation of GRP78 was clearly seen in *ZNF313*^*+*^ but not in *ZNF313*^*-*^ subline of the HAP1 haploid human cell line, further supporting the ZNF313 dependency of XAF1 regulation of GRP78 (Fig. 4I). As predicted, XAF1’s function to promote TG-mediated apoptosis was impeded in ZNF313-depleted cells (Fig. 4J and Supplementary Fig. 4E).

### *XAF1* transcription is activated by ER stress through PERK-Nrf2 signaling

**Fig. 5:**
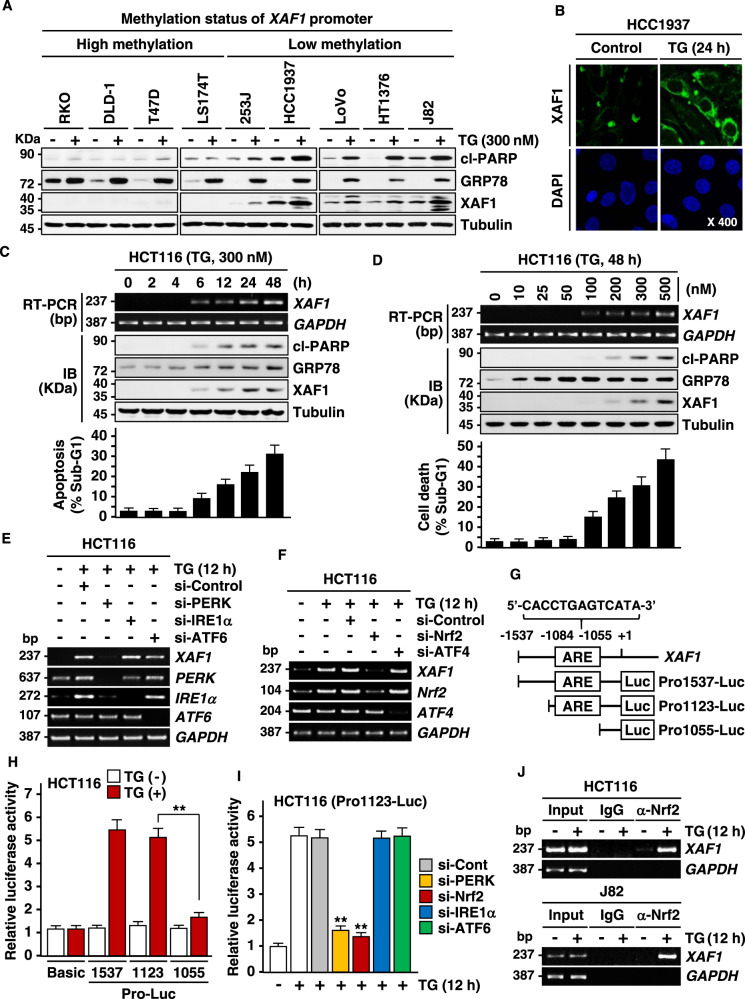
*XAF1* transcription is activated by ER stress via PERK-Nrf2 signaling. **A** XAF1 upregulation by TG and its association with GRP78 expression and apoptosis induction in human cell lines. **B** Immunofluorescence microscopic analysis of XAF1 in HCC1937 cells. Cells were treated with DMSO (control) or TG (300 nM) for 24 h. DAPI was used for counterstaining of the nuclei. **C** A time kinetics of *XAF1* mRNA induction following TG exposure and its association with apoptosis induction. **D** Induction of *XAF1* mRNA expression by cytotoxic doses of TG. **E**, **F** Disruption of TG induction of *XAF1* mRNA by depletion of PERK or Nrf2. Cells were transfected with 50 pM of siRNAs as indicated and then exposed to TG (300 nM) for 12 h. **G** A putative ARE in the *XAF1* promoter and reporter construction for luciferase assay. Luc, luciferase. **H**, **I** TG activation of the reporter containing the putative ARE and its attenuation by depletion of PERK or Nrf2. Cells were transfected with the reporters and then exposed to TG (300 nM) for 12 h. **J** ChIP assays showing Nrf2 binding to the ARE in TG-treated cells. Cells were exposed to TG (300 nM) for 12 h. α-Nrf2, Nrf2-specific antibody. Data represent the mean ± SD of triplicate assays. **P < 0.01 (Student *t*-test).

Our results have shown that expression status of XAF1 is associated with the apoptotic sensitivity to ER stress, suggesting that loss or downregulation of XAF1 by promoter hypermethylation may result in GRP78 elevation, thereby enhancing tumor cell resistance to ER stress (Fig. 5A, B and Supplementary Fig. 5A). Meanwhile, it was also recognized that XAF1 expression is induced by ER stress to drive apoptotic switch of UPR function (Fig. 2A). To further address this issue, we characterized signaling pathway responsible for the ER stress-mediated XAF1 induction. *XAF1* mRNA expression was upregulated by TG, TM, and BFA and this induction was abrogated by pretreatment of actinomycin D (Act D), an inhibitor of *de novo* RNA synthesis, indicating the transcriptional activation of *XAF1* by ER stress (Supplementary Fig. 5B, C). Assays using increasing treatment times and doses showed that XAF1 induction is linked to apoptotic response to TG, suggesting that XAF1 may act as a switch in cell-fate decisions under ER stress conditions (Fig. 5C, D and Supplementary Fig. 5D, E). In addition, we examined effect of XAF1 induction on GRP78, which is also upregulated by ER stress. Compared to J82-*XAF1*^*+/+*^ cells, J82-*XAF1*^*−/−*^ cells displayed a delayed induction kinetics but higher protein level of GRP78 (Supplementary Fig. 5F, G). As predicted, an increased interaction was detected between the upregulated XAF1 and GRP78 proteins (Supplementary Fig. 5H). It was also shown that blockade of TG induction of XAF1 results in GRP78 elevation, verifying the GRP78-degarading effect of activated XAF1 under ER stress conditions (Supplementary Fig. 5I). *XAF1* induction by TG was strongly impeded by depletion of PERK or its inhibitor GSK2606414 but not affected by depletion of IRE1α or ATF6 (Fig. 5E and Supplementary Fig. 5J, K). It was also blocked by depletion of a PERK effector nuclear factor erythroid 2-related factor 2 (Nrf2) but not affected by another effector ATF4, indicating that *XAF1* transcription is activated through PERK-Nrf2 signaling (Fig. 5F and Supplementary Fig. 5L). Consistently, *XAF1* mRNA was induced by PERK overexpression, and PERK activation of XAF1 was abolished if Nrf2 is depleted (Supplementary Fig. 5M). We identified a putative Nrf2 response element (ARE) in the 5′ upstream region (nucleotides -1055 to -1084 relative to ATG) of the *XAF1* gene (Fig. 5G). The activity of reporters containing this ARE (Pro1537 and Pro1123) was strongly activated by TG treatment while a reporter omitting the ARE (Pro1055) showed no response to TG (Fig. 5H). The Pro1123 reporter response to TG was significantly decreased by depletion of either PERK or Nrf2 but not affected by depletion of IRE1α or ATF6 (Fig. 5I and Supplementary Fig. 5N). A chromatin immunoprecipitation (ChIP) assay showed that the ARE within the *XAF1* promoter is occupied directly by Nrf2 (Fig. 5J). These indicate that *XAF1* is a bona fide transcription target of Nrf2 whose activation drives apoptotic ER stress response.

### XAF1 blocks CHIP-IRE1α axis to direct apoptotic switch of ER stress response

**Fig. 6:**
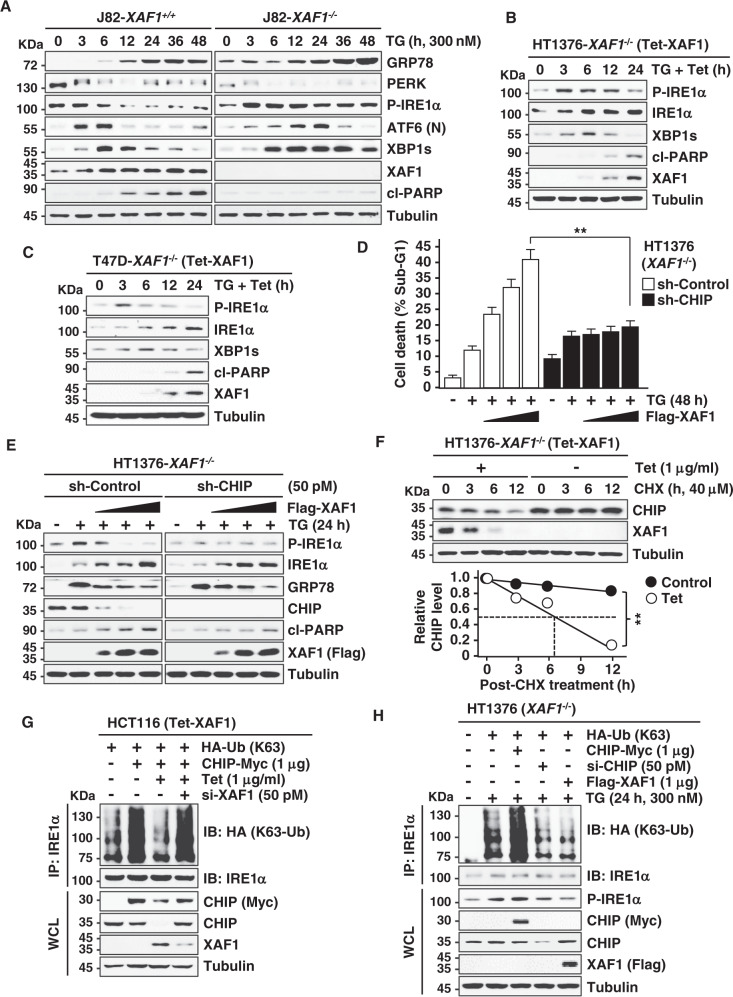
XAF1 inhibits IRE1α phosphorylation by destabilizing CHIP. **A** Comparison of TG activation of three UPR transducers in *XAF1*^*+/+*^ and *XAF1*^*−/−*^ sublines of J82 cells. **B**, **C** Effect of XAF1 expression on TG-induced IRE1α phosphorylation and XBP1s expression in *XAF1*^*−/−*^ cells. Cells were treated with TG (300 nM, 36 h) and XAF1 induction was achieved by Tet-inducible XAF1 (Tet-XAF1) system. The TG-treated cells were incubated with tetracycline (1 μg/ml) as indicated. **D**, **E** A CHIP dependency of XAF1 effect on TG-induced IREα phosphorylation and apoptosis. Cells were treated with TG (300 nM). **F** CHX chase experiment showing XAF1 destabilization of CHIP. XAF1 induction was achieved by Tet-inducible XAF1 (Tet-XAF1) system. Cells were incubated with CHX (40 μM) for indicated hours. **G**, **H** Effect of XAF1 expression on CHIP or TG-induced K63-linked ubiquitination of IREα.

ER stress response is determined mainly by UPR signaling pathways [3]. We assessed whether XAF1 drives ER stress-mediated apoptosis through the regulation of UPR signalings. Following TG treatment, IRE1α displayed much higher and prolonged phosphorylation in J82-*XAF1*^*−/−*^ compared to J82-*XAF1*^*+/+*^ subline while PERK and ATF6 showed delayed activation kinetics in *XAF1*^*−/*^^−^ sublines (Fig. 6A and Supplementary Fig. 6A). In J82-*XAF1*^*+/+*^ cells, XAF1 induction was apparent at 6h after TG treatment and associated with a decline of IRE1α phosphorylation and induction of apoptosis. IRE1α activation is known to associate with adaptive response and reported to attenuate under irresolvable ER stress to induce apoptosis [34, 35]. IRE1α affects several biological processes through its endoribonuclease activity that carries out an unconventional splicing of target mRNAs, including *XBP1 and BLOC1S1* [36–38]. We observed that TG-mediated IRE1α phosphorylation and cleavage of IRE1α target mRNAs (*XBP1s* and *BLOC1S1*) are reinforced by XAF1 depletion, supporting that XAF1 attenuates IRE1α endoribonuclease activity to promote apoptosis (Supplementary Fig. 6B). Assays utilizing *XAF1*^−/−^ sublines (HT1376 and T47D) with the Tet-inducible XAF1 also showed the XAF1-mediated decline of TG-induced IRE1α phosphorylation and XBP1s expression (Fig. 6B, C). The E3 ligase C-terminus of Hsc70-interacting protein (CHIP) increases IRE1α phosphorylation through K63-linked ubiquitination, which is associated with adaptive response to ER stress and cell survival [39]. Consistently, we observed that TG-induced IRE1α phosphorylation is markedly attenuated in CHIP-depleted cells (Supplementary Fig. 6C). Based on our recent study demonstrating that XAF1 downregulates CHIP expression, we tested whether XAF1 inhibits IRE1α phosphorylation through CHIP regulation [29]. Transient transfection or Tet-mediated induction of XAF1 led to a drastic reduction of CHIP protein and its effects on TG-induced IRE1α phosphorylation and apoptosis were profoundly impaired in CHIP-depleted cells (Fig. 6D, E and Supplementary Fig. 6D–G). As predicted, the half-life of CHIP protein was substantially shortened by XAF1 induction and this effect was blocked by MG132, supporting that XAF1 induces the proteasomal degradation of CHIP (Fig. 6F and Supplementary Fig. 6H). Finally, we asked if XAF1 suppresses CHIP-mediated K63-linked ubiquitination of IRE1α. IP assay revealed that XAF1 blocks K63-linked ubiquitination of IRE1α triggered by TG treatment or CHIP transfection (Fig. 6G, H and Supplementary Fig. 6I). These indicate that XAF1 activation directs an apoptotic switch of ER stress response by blocking the adaptive CHIP-IRE1α axis.

### XAF1 enhances therapeutic effect of ER stress inducer in vivo

**Fig. 7:**
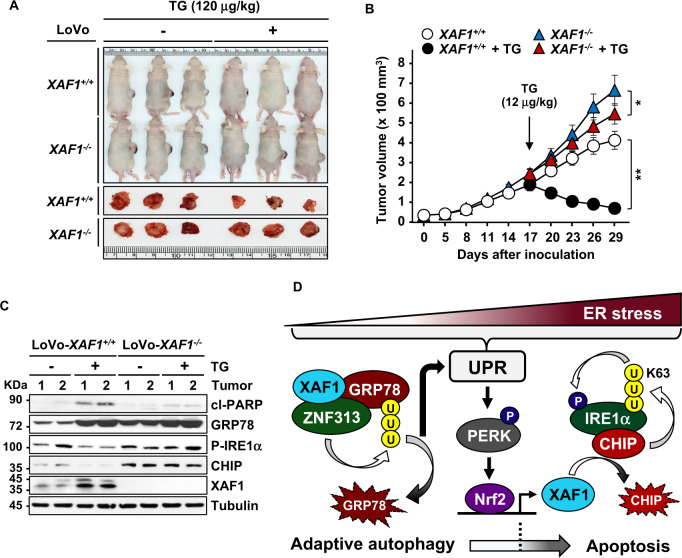
XAF1 enhances tumor response to cytotoxic ER stress. **A**, **B** Mouse tumor xenograft assay showing XAF1 effect on tumor response to TG. Tumors were derived from *XAF1*^*+/+*^ and *XAF1*^*−/*^^−^ sublines of LoVo cells and exposed to saline or TG (12 μg/kg) at day 17 by intratumoral injection. Representative photographs of xenograft tumors at day 29 were shown. Data represent the mean ± SD (n = 6 per group). *P < 0.05; **P < 0.01 (Student *t*-test). **C** IB assays of GRP78, IRE1α, CHIP in xenograft tumor tissues. **D** Schematic representation of the molecular mechanism underlying XAF1-driven apoptotic switch of ER stress response.

To investigate the XAF1 role in tumor response to TG in vivo, we carried out mouse tumor xenograft assays using *XAF1*^*+/+*^ and *XAF1*^*−/−*^ sublines of LoVo cells. Compared to *XAF1*^*+/+*^ tumors, *XAF1*^*−/−*^ tumors displayed an increased growth rate (Fig. 7A, B). In response to a cytotoxic dose of TG (12 μg/kg), *XAF1*^−^^*/−*^ tumors showed much lower regression compared to *XAF1*^*+/+*^ tumors (16.7% versus 78.6%). IB assay of xenograft tissues revealed that *XAF1*^*−/−*^ tumors have higher GRP78, CHIP, and phosphorylated IRE1α levels compared to *XAF1*^*+/+*^ tumors (Fig. 7C). Collectively, our study identifies XAF1 as a key regulator of ER stress response, illuminating its novel role as a tumor suppressor and the mechanistic consequence of its epigenetic alteration in tumorigenesis (Fig. 7D).

## Discussion

Despite accumulating evidence supporting that XAF1 is activated under various stressful conditions and directs an apoptotic switch of a stress response, its role in ER stress response has not been studied yet. In the present study, we identified that XAF1 increases apoptotic sensitivity to ER stress and dictates the outcomes of UPR signaling through the modulation of the ER stress sensor GRP78 and the UPR signal transducer IRE1α. Therefore, our study demonstrates that XAF1 functions as a linchpin to govern the cell-fate decisions under ER stress conditions favoring apoptosis over adaptation.

GRP78 is the most abundant and major chaperone in the ER, which acts as an ER stress sensor that detects ER imbalance and fine-tunes the threshold for UPR initiation [5, 40]. GRP78 upregulation in multiple human tumors contributes to tumor cell survival and chemoresistance through high tolerance for ER stress while its suppression leads to enhanced cytotoxic response to chemotherapeutic drugs [13–16, 41]. Increased GRP78 expression is associated with higher pathological grades, high risk of recurrence, and poor prognosis of cancer patients [41–45]. Therefore, GRP78 has been suggested as a target for therapeutic intervention. Studies demonstrated that GRP78 is destabilized by various tumor suppressors while it is stabilized by oncogenic factors [42, 44, 46–48]. Nevertheless, the molecular mechanisms underlying GRP78 upregulation in the tumorigenic process have not been well understood. In the current study, we present evidence that XAF1 directly interacts with and destabilizes GRP78 to enhance apoptotic cellular response to ER stress. We also observed an inverse correlation of GRP78 and XAF1 expression in human cancer cell lines of various origins and primary breast carcinoma tissues. Given that loss or reduction of *XAF1* expression by aberrant promoter hypermethylation is common in human cancers, our data support that epigenetic silencing of *XAF1* is a mechanism leading to abnormal elevation of GRP78 and tumor resistance to ER stress.

Upon GRP78 dissociation, UPR signaling transducers IRE1 and PERK are activated through dimerization and autophosphorylation [3]. IRE1 splices *XBP1* mRNA to generate XBP1s, which improves stress adaptation by transactivating genes involved in the ER protein quality control. PERK inhibits global protein synthesis through phosphorylation of eIF2α to reduce ER protein loading. Under prolonged or irreversible ER stress conditions, PERK activity is sustained, while IRE1 phosphorylation is turned off [34, 35]. Attenuated IRE1 signaling dissolves its oligomers and reduces RNase activity, which eventually makes cells sensitive to death [49]. The Nrf2 transcription factor, one of phosphorylation substrates of PERK, activates multiple target genes that are involved mainly in cytoprotective response to stress [50, 51]. Nrf2 activation increases expression of proteasome-related genes and anti-apoptotic proteins, such as Bcl-2 and Bcl-xL to enhance cell survival and drug resistance [52–54]. Intriguingly, we identified *XAF1* as a bona fide transcription target of Nrf2 under ER stress conditions. It is thus plausible that Nrf2 might be modified differentially by cytostatic and cytotoxic stresses, allowing its selective or preferential binding to a subset of target promoters and thereby provoking both adaptive and apoptotic effects depending on its target selectivity. Given that tumor cells with *XAF1* hypermethylation require high dose of stress inducers for XAF1 activation, promoter methylation status of *XAF1* could be a predictive marker for the clinical efficacy of ER stress-based therapy. This is supported by the finding that blockade of XAF1 induction disrupts tumor regression by treatment of ER stress inducer in vivo. Considering that Nrf2 and XAF1 are up- and downregulated, respectively in many human cancers, alteration of the Nrf2-XAF1 axes might contribute to tumor resistance to ER stress. Together, our study establishes XAF1 as a key target of UPR in signaling apoptosis, suggesting that its alteration might be a critical event in the enhanced adaptation function of UPR signaling and the appearance of ER stress resistance in tumorigenesis.

Regulation of IRE1α is essential for the cell-fate decisions under ER stress conditions. IRE1α is initially phosphorylated via K63-linked ubiquitination by CHIP to exert its adaptation function and dephosphorylated during the terminal phase of UPR by RPAP2 phosphatase [39, 55]. The CHIP-IRE1α axis is associated with adaptive response and cell survival, and IRE1α activity attenuates under irresolvable ER stress to induce apoptosis [34, 35, 39]. However, the molecular mechanism of IRE1 regulation has been poorly understood. In this study, we identified that XAF1 destabilizes CHIP to suppress K63 ubiquitination of IRE1α, suggesting that XAF1 may induce the proteasomal degradation of CHIP through the assembly of an E3 ligase-mediated destruction complex. Considering that K63 ubiquitination serves as a scaffold for protein–protein interaction and regulates cellular localization of proteins, it is conceivable that XAF1 may affect IRE1α binding to proteins and its ER membrane localization [56]. IRE1 has been known to influence multiple biological processes through its endoribonuclease activity that is involved in unconventional splicing of several mRNAs, such as *XBP1* and *BLOC1S1* [36–38]. We found that XAF1 activation blocks TG induction of *XBP1s*, which is known as a pro-survival factor, supporting that XAF1 affects the endoribonuclease activity of IRE1α. It is thus plausible that XAF1 accelerates ER stress-mediated apoptosis at least partially by attenuating the adaptive function of the IRE1α-XBP1s axis. Considering that the endoribonuclease activity of IRE1α can evoke pro-apoptotic effect, further molecular studies will be required to understand whether XAF1 regulation of the CHIP-IRE1α axis can have a selective modulation effect on the endoribonuclease activity of IRE1α, including the selection of target mRNAs.

Collectively, this study uncovers that XAF1 represents one crucial molecular switch in ER stress-induced UPR-mediated cell-fate decisions, adding a new layer of complexity to the mechanisms by which the outcomes of UPR signaling is determined. Our study uncovers a novel tumor suppressive role of XAF1, raising the possibility that the restoration of the XAF1-GRP78 and/or XAF1-CHIP-IRE1α interplay could be an attractive avenue for the therapeutic intervention of tumor progression.

## Materials and methods

Details regarding cell culture and reagents, knockout cell lines, expression plasmids and siRNAs, luciferase reporter activity assay, semi-quantitative reverse transcription–polymerase chain reaction (RT-PCR), ChIP, immunoblot, immunoprecipitation, immunohistochemistry, immunofluorescence, GST pull-down and in vitro binding assays, ubiquitination assay, growth and apoptosis assay, animal studies, and statistical analysis are available in supplementary Materials and Methods. All studies were performed with the approval of Korea University Institutional Animal Care and Use Committee and Korea Animal Protection Law.

## Supplementary information

Supplementary Figures

Supplementary information-Materials and Methods & SFigure legends

Densitometric measurement of Western blots

Western blots - Uncropped

aj-checklist

## Data Availability

All data generated or analyzed during this study are included in this published article and its supplementary information files.
