# Supplementary material for: XAF1 drives apoptotic switch of endoplasmic reticulum stress response through destabilization of GRP78 and CHIP
Source: Cell Death Dis. 2022 Jul 28;13(7):655. doi: 10.1038/s41419-022-05112-0 (PMC9334361; doi:10.1038/s41419-022-05112-0)
Supplement: Supplementary file 1 — Supplementary Figures [file 41419_2022_5112_MOESM1_ESM.pptx]

## Slide 1
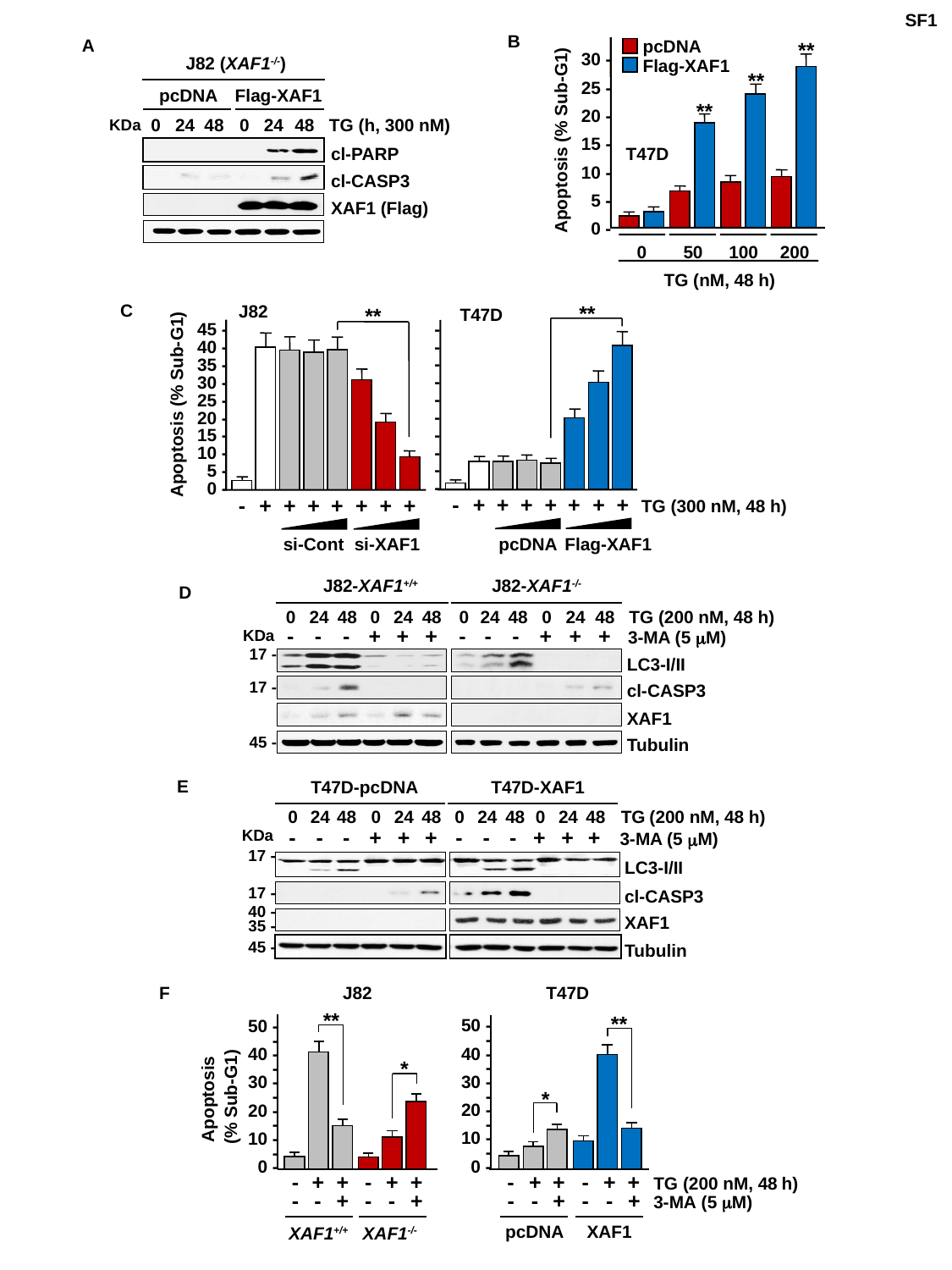

SF1
B
A
**
pcDNA
Flag-XAF1
30 -
25 -
20 -
15 -
10 -
5 -
0 -
**
**
Apoptosis (% Sub-G1)
T47D
 0 50 100 200
 TG (nM, 48 h)
J82 (XAF1-/-)
 pcDNA Flag-XAF1
0 24 48 0 24 48 TG (h, 300 nM)
KDa
cl-PARP
cl-CASP3
XAF1 (Flag)
Tubulin
90 -
17 -
40 -
35 -
45 -
J82
T47D
**
**
 -
-
 -
 -
 -
 -
 -
 -
 -
 -
45 -
40 -
35 -
30 -
 25 -
20 -
 15 -
10 -
 5 -
0 -
Apoptosis (% Sub-G1)
- + + + + + + + TG (300 nM, 48 h)
- + + + + + + +
pcDNA Flag-XAF1
si-Cont si-XAF1
C
J82-XAF1+/+ J82-XAF1-/-
0 24 48 0 24 48 0 24 48 0 24 48 TG (200 nM, 48 h)
- - - + + + - - - + + + 3-MA (5 μM)
KDa
LC3-I/II
cl-CASP3
XAF1
Tubulin
17 -
17 -
40 -
35 -
45 -
D
E
T47D-pcDNA T47D-XAF1
0 24 48 0 24 48 0 24 48 0 24 48 TG (200 nM, 48 h)
- - - + + + - - - + + + 3-MA (5 μM)
KDa
17 -
17 -
40 - 35 -
45 -
LC3-I/II
cl-CASP3
XAF1
Tubulin
F
J82
T47D
**
**
50 -
 -
40 -
 -
30 -
 -
20 -
 -
10 -
 -
0 -
50 -
 -
40 -
 -
30 -
 -
20 -
 -
10 -
 -
0 -
*
Apoptosis
(% Sub-G1)
*
- + + - + + - + + - + + TG (200 nM, 48 h)
- - + - - + - - + - - + 3-MA (5 μM)
pcDNA XAF1
XAF1+/+ XAF1-/-

## Slide 2
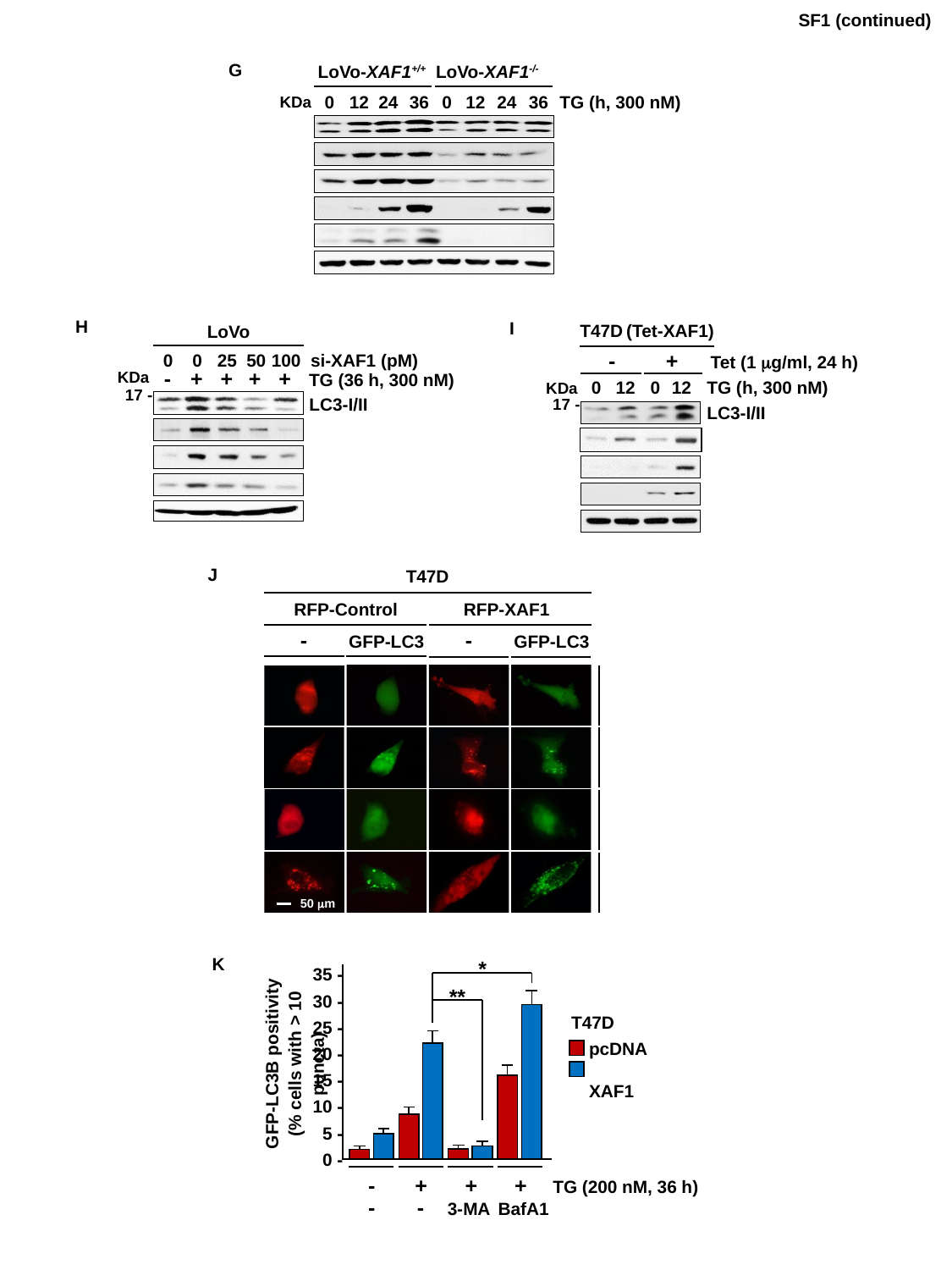

SF1 (continued)
G
LoVo-XAF1+/+ LoVo-XAF1-/-
0 12 24 36 0 12 24 36 TG (h, 300 nM)
KDa
LC3-I/II
Beclin1
Atg5-Atg12
cl-PARP
XAF1
Tubulin
17 -
60 -
50 -
90 -
40 -
35 -
45 -
H
I
LoVo
0 0 25 50 100 si-XAF1 (pM)
KDa
- + + + + TG (36 h, 300 nM)
17 -
60 -
90 -
40 -
35 -
45 -
LC3-I/II
Beclin-1
cl-PARP
XAF1
Tubulin
T47D (Tet-XAF1)
- + Tet (1 μg/ml, 24 h)
0 12 0 12 TG (h, 300 nM)
KDa
17 -
60 -
90 -
40 -
35 -
45 -
LC3-I/II
Beclin-1
cl-PARP
XAF1
Tubulin
J
T47D
RFP-Control RFP-XAF1
 GFP-LC3
- - GFP-LC3
Control
TG (200 nM)
TG +
3-MA (5 μM)
TG +
BafA1 (100 nM)
50 μm
*
35 -
30 -
25 -
20 -
15 -
10 -
5 -
0 -
**
T47D
pcDNA
XAF1
GFP-LC3B positivity
(% cells with > 10 puncta)
- + + + TG (200 nM, 36 h)
- - 3-MA BafA1
K

## Slide 3
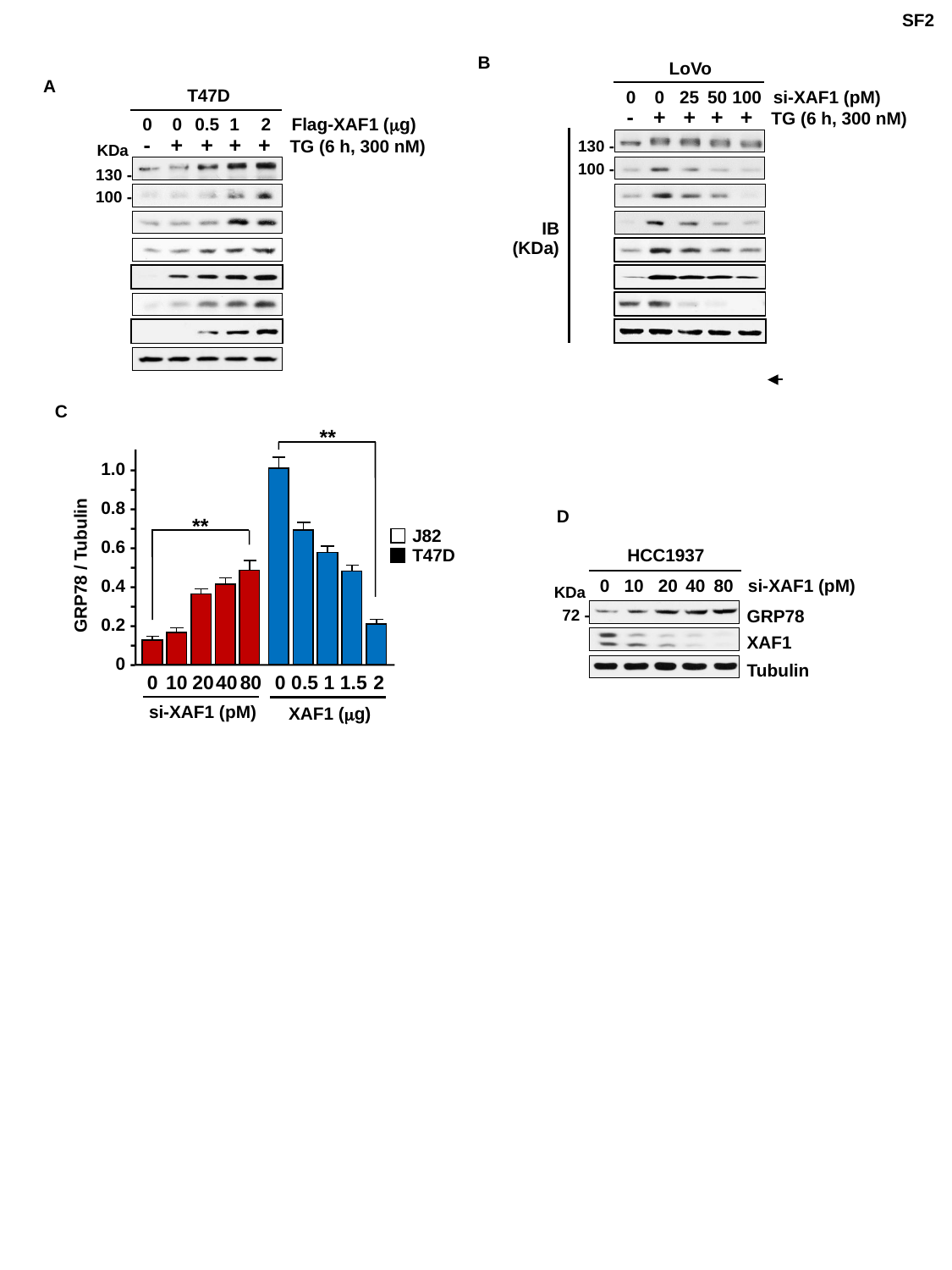

SF2
B
LoVo
0 0 25 50 100 si-XAF1 (pM)
- + + + + TG (6 h, 300 nM)
PERK
P-IRE1α
ATF6 (N)
ATF4
P-eIF2α
CHOP
XAF1
Tubulin
130 -
100 -
55 -
43 -
40 -
35 -
25 -
40 -
35 -
45 -
IB
(KDa)
GAPDH
A
T47D
0 0 0.5 1 2 Flag-XAF1 (μg)
- + + + + TG (6 h, 300 nM)
KDa
PERK
P-IRE1α
ATF6 (N)
ATF4
P-eIF2α
CHOP
XAF1 (Flag)
Tubulin
130 -
100 -
55 -
43 -
40 -
35 -
25 -
40 -
35 -
45 -
C
**
 1.0 -
-
0.8 -
-
0.6 -
-
0.4 -
-
0.2 -
 -
0 -
**
J82
T47D
GRP78 / Tubulin
0 10 20 40 80 0 0.5 1 1.5 2
XAF1 (μg)
 si-XAF1 (pM)
D
HCC1937
0 10 20 40 80 si-XAF1 (pM)
KDa
GRP78
XAF1
Tubulin
72 -
45 -
35 -
45 -

## Slide 4
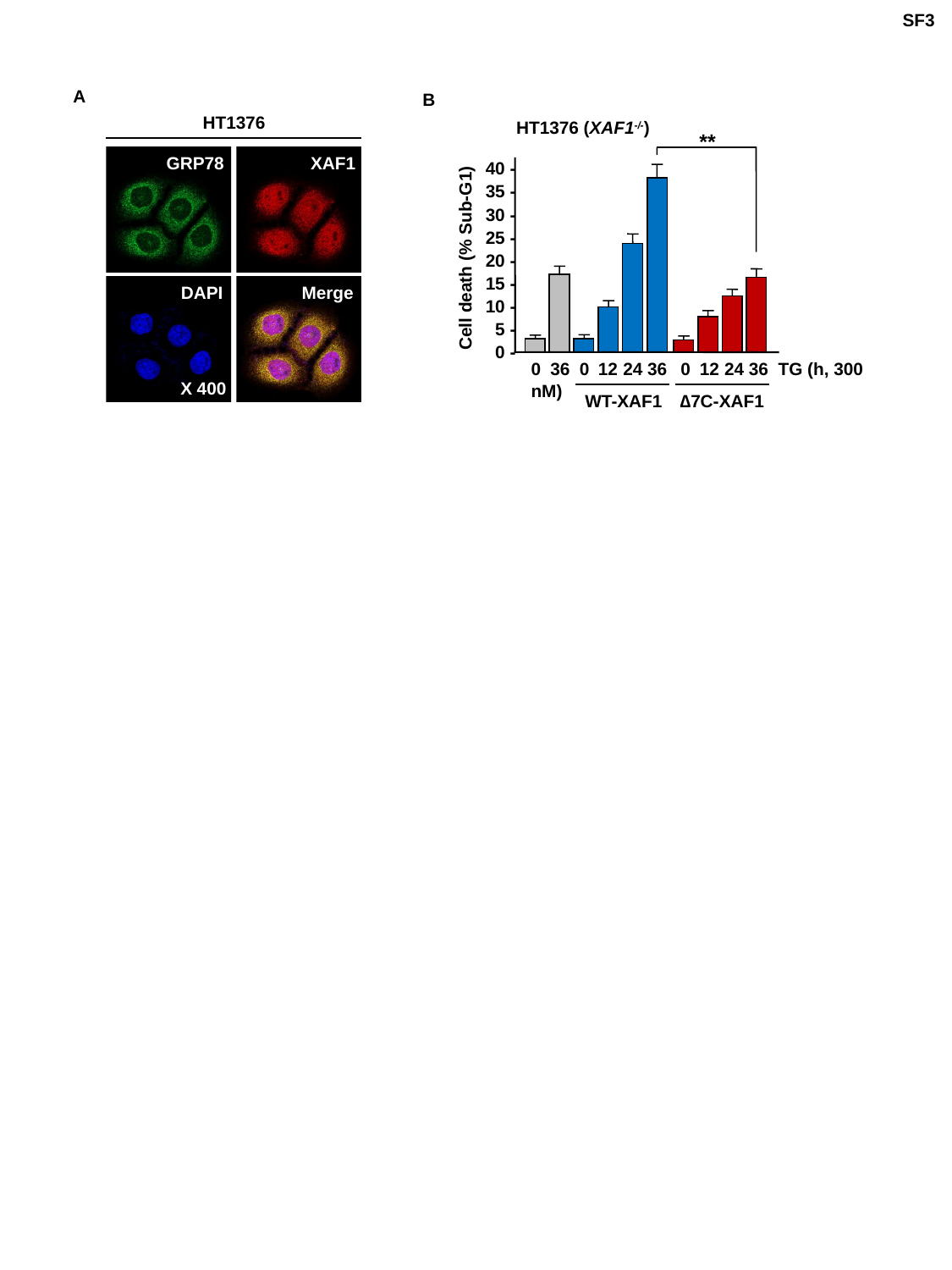

SF3
A
B
HT1376
GRP78 XAF1
 DAPI Merge
X 400
HT1376 (XAF1-/-)
**
40 -35 -
30 -
25 -
20 -
15 -
10 -
5 -
0 -
Cell death (% Sub-G1)
0 36 0 12 24 36 0 12 24 36 TG (h, 300 nM)
WT-XAF1 ∆7C-XAF1

## Slide 5
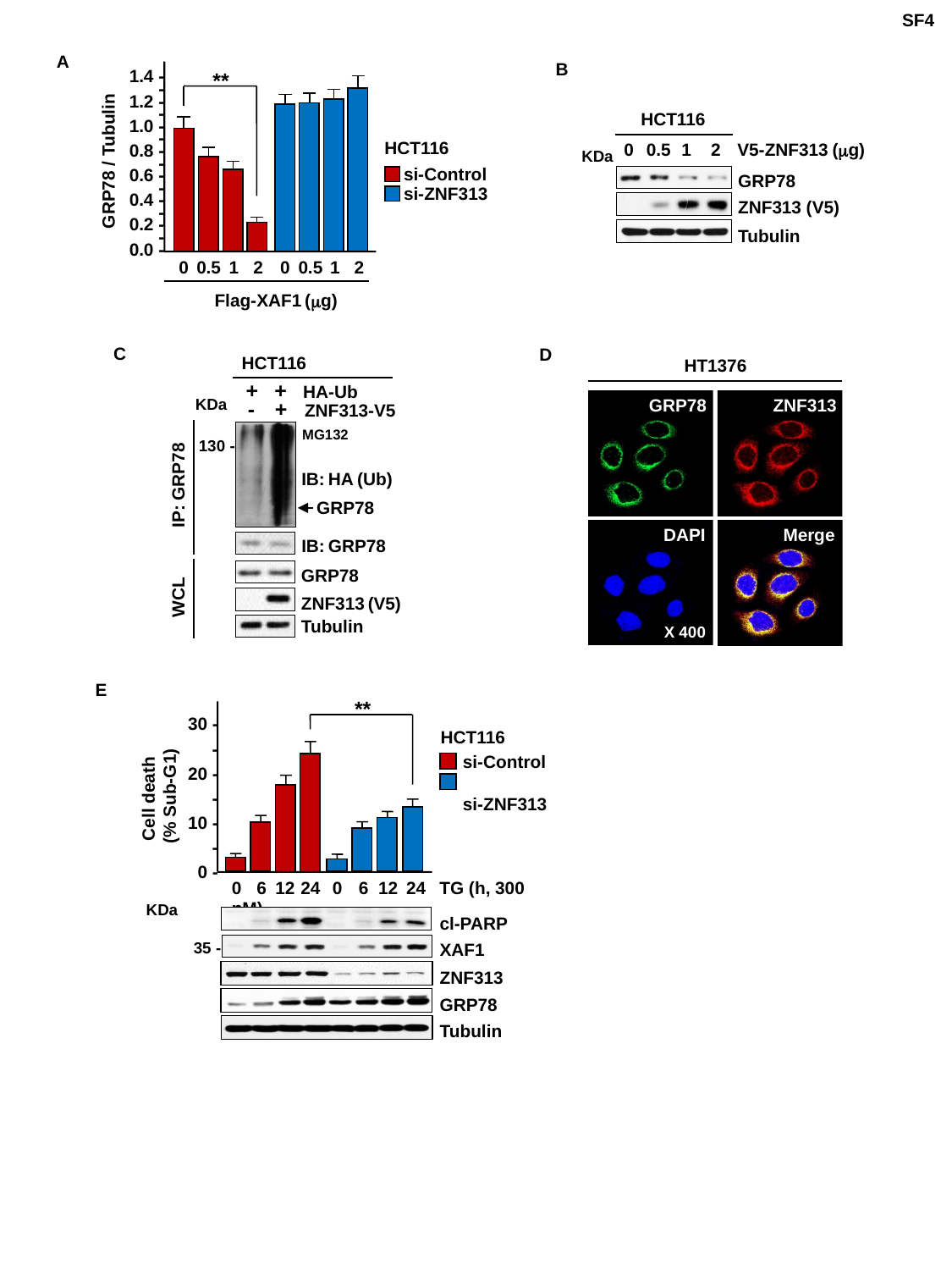

SF4
A
B
**
 1.4 -
-
1.2 -
-
1.0 -
-
0.8 -
-
0.6 -
-
0.4 -
-
0.2 -
 -
0.0 -
HCT116
GRP78 / Tubulin
si-Control si-ZNF313
0 0.5 1 2 0 0.5 1 2
 Flag-XAF1 (μg)
HCT116
0 0.5 1 2 V5-ZNF313 (μg)
KDa
72 -
35 -
45 -
GRP78
ZNF313 (V5)
Tubulin
C
D
HCT116
+ + HA-Ub
KDa
- + ZNF313-V5
170 -
130 -
100 -
72 -
72 -
72 -
35 -
45 -
MG132
IB: HA (Ub)
IB: GRP78
 IP: GRP78
GRP78
GRP78
ZNF313 (V5)
Tubulin
WCL
HT1376
GRP78 ZNF313
 DAPI Merge
X 400
E
**
30 -
 -
20 -
 -
10 -
 -
0 -
HCT116
si-Control
si-ZNF313
Cell death
(% Sub-G1)
0 6 12 24 0 6 12 24 TG (h, 300 nM)
KDa
cl-PARP
XAF1
ZNF313
GRP78
Tubulin
90 -
45 -
35 -
35 -
72 -
45 -

## Slide 6
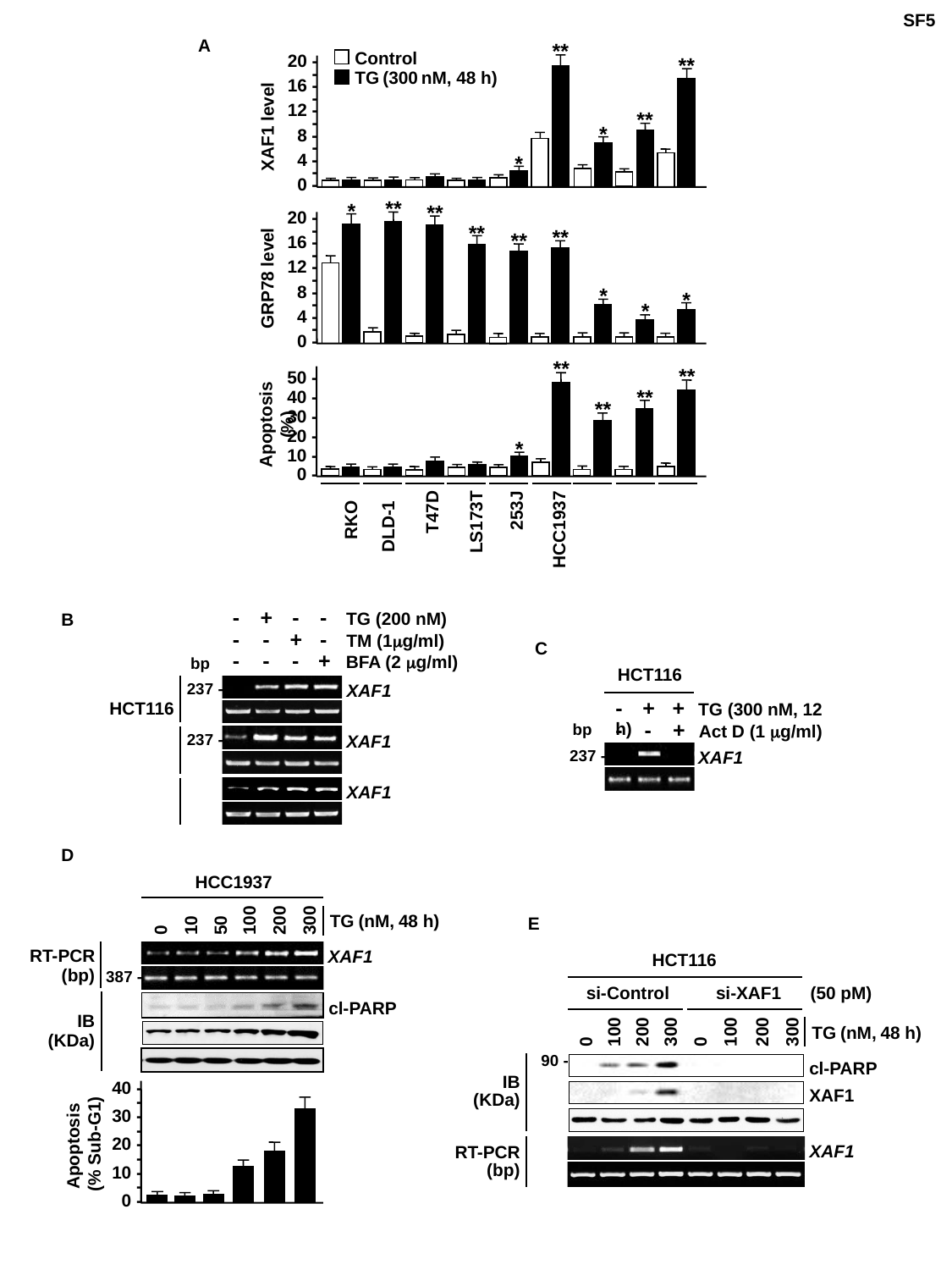

SF5
A
**
Control
TG (300 nM, 48 h)
**
20 -
-
16 -
 -
12 -
 -
8 -
-
4 -
-
0 -
**
XAF1 level
*
*
**
*
**
20 -
-
16 -
 -
12 -
 -
8 -
-
4 -
-
0 -
**
**
**
GRP78 level
*
*
*
**
50 -
40 -
30 -
20 -
10 -
0 -
**
**
**
RKO
DLD-1
T47D
LS173T
253J
HCC1937
Apoptosis (%)
*
LoVo
HT1376
J82
B
- + - - TG (200 nM)
- - + - TM (1μg/ml)
- - - + BFA (2 μg/ml)
bp
HCT116
J82
HT1376
237 -
387 -
237 -
387 -
237 -
387 -
XAF1
GAPDH
XAF1
GAPDH
XAF1
GAPDH
C
HCT116
- + + TG (300 nM, 12 h)
bp
- - + Act D (1 μg/ml)
237 -
387 -
XAF1
GAPDH
D
0
10
50
100
200
300
HCC1937
TG (nM, 48 h)
XAF1
GAPDH
RT-PCR (bp)
IB
(KDa)
237 -
387 -
cl-PARP
XAF1
Tubulin
90 -
40 -
35 -
45 -
40 -
 -
30 -
 -
20 -
 -
10 -
 -
0 -
Apoptosis
(% Sub-G1)
E
0
100
200
300
0
100
200
300
HCT116
si-Control si-XAF1 (50 pM)
TG (nM, 48 h)
90 -
40 -
35 -
45 -
237 -
387 -
cl-PARP
XAF1
Tubulin
IB
(KDa)
RT-PCR
(bp)
XAF1
GAPDH

## Slide 7
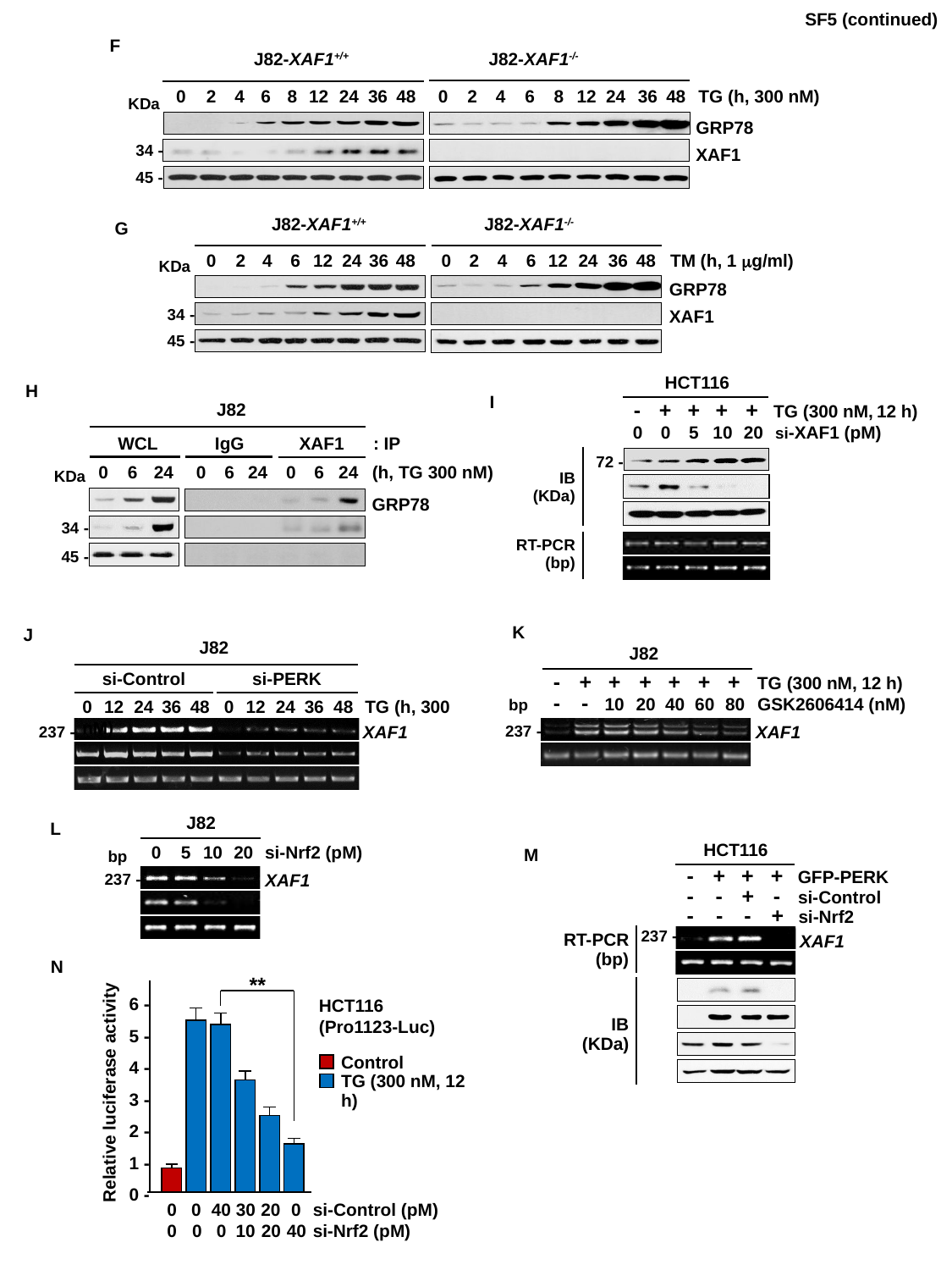

SF5 (continued)
F
J82-XAF1+/+ J82-XAF1-/-
0 2 4 6 8 12 24 36 48 0 2 4 6 8 12 24 36 48 TG (h, 300 nM)
KDa
GRP78
XAF1
Tubulin
72 -
34 -
45 -
G
J82-XAF1+/+ J82-XAF1-/-
0 2 4 6 12 24 36 48 0 2 4 6 12 24 36 48 TM (h, 1 μg/ml)
KDa
GRP78
XAF1
Tubulin
72 -
34 -
45 -
HCT116
 - + + + + TG (300 nM, 12 h)
0 0 5 10 20 si-XAF1 (pM)
72 -
45 -
35 -
45 -
331 -
387 -
GRP78
XAF1
Tubulin
IB
(KDa)
RT-PCR
(bp)
GRP78
GAPDH
H
I
J82
WCL IgG XAF1 : IP
0 6 24 0 6 24 0 6 24 (h, TG 300 nM)
KDa
GRP78
XAF1
Tubulin
72 -
34 -
45 -
K
J
J82
si-Control
si-PERK
0 12 24 36 48 0 12 24 36 48 TG (h, 300 nM)
237 -
637 -
387 -
XAF1
PERK
GAPDH
J82
- + + + + + + TG (300 nM, 12 h)
bp
- - 10 20 40 60 80 GSK2606414 (nM)
237 -
387 -
XAF1
GAPDH
J82
0 5 10 20 si-Nrf2 (pM)
bp
237 -
104 -
387 -
XAF1
Nrf2
GAPDH
L
HCT116
- + + + GFP-PERK
- - + - si-Control
- - - + si-Nrf2
XAF1
GAPDH
XAF1
PERK (GFP)
Nrf2
Tubulin
237 -
387 -
40 -
35 -
130 -
60 -
45 -
RT-PCR
(bp)
IB
(KDa)
M
N
**
6 -
5 -
4 -
3 -
2 -
1 -
0 -
HCT116
(Pro1123-Luc)
Control
TG (300 nM, 12 h)
Relative luciferase activity
0 0 40 30 20 0 si-Control (pM)
0 0 0 10 20 40 si-Nrf2 (pM)

## Slide 8
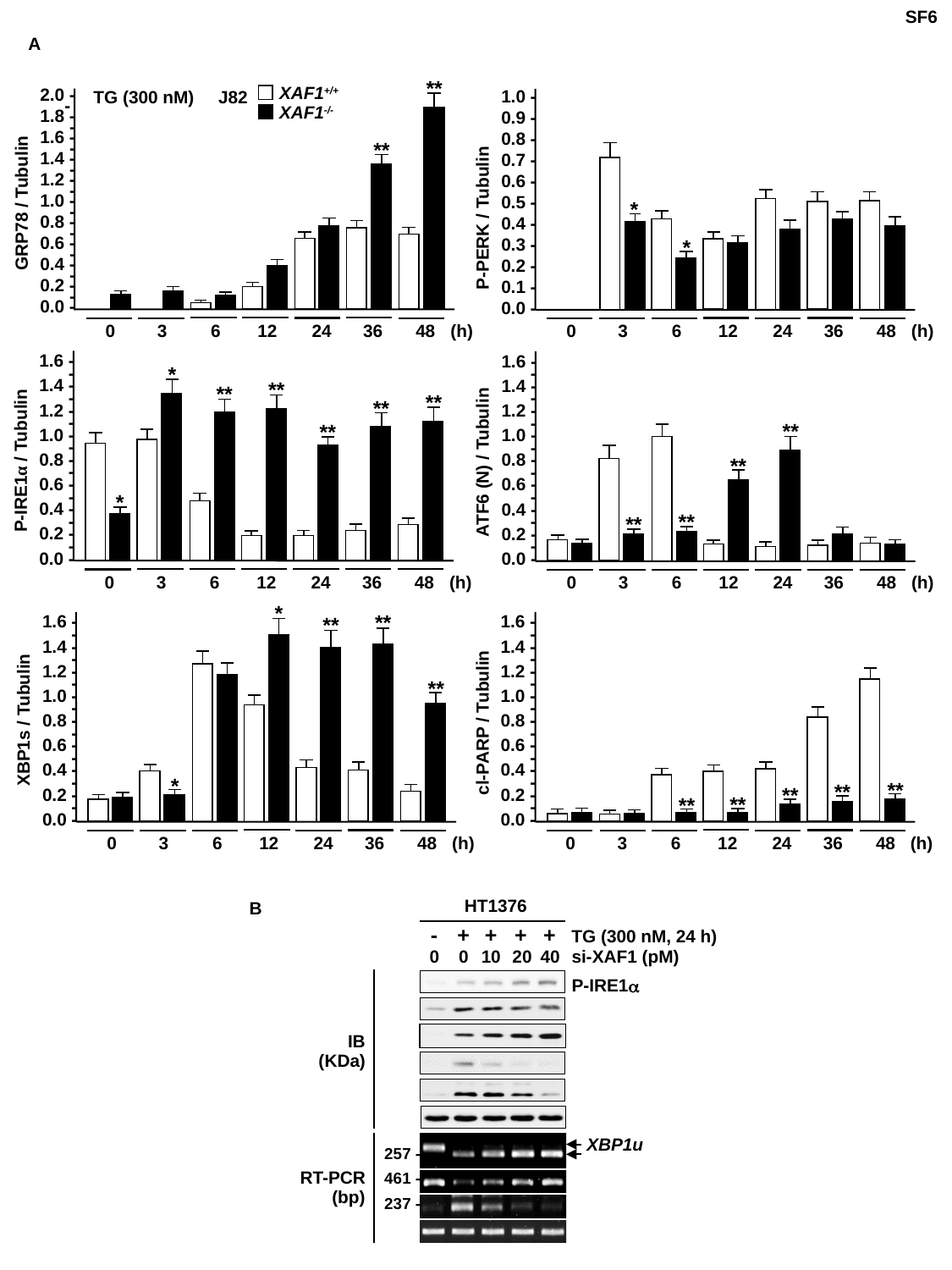

SF6
A
TG (300 nM)
XAF1+/+
XAF1-/-
J82
**
 2.0 -
-
1.8 -
-
1.6 -
-
1.4 -
-
1.2 -
-
 1.0 -
-
0.8 -
-
0.6 -
-
0.4 -
-
0.2 -
 -
0.0 -
**
GRP78 / Tubulin
0 3 6 12 24 36 48 (h)
 1.0 -
0.9 -
0.8 -
0.7 -
0.6 -
0.5 -
0.4 -
0.3 -
0.2 -
 0.1 -
0.0 -
*
P-PERK / Tubulin
*
0 3 6 12 24 36 48 (h)
 1.6 -
-
1.4 -
-
1.2 -
-
 1.0 -
-
0.8 -
-
0.6 -
-
0.4 -
-
0.2 -
 -
0.0 -
*
**
**
**
**
**
P-IRE1α / Tubulin
*
0 3 6 12 24 36 48 (h)
 1.6 -
-
1.4 -
-
1.2 -
-
 1.0 -
-
0.8 -
-
0.6 -
-
0.4 -
-
0.2 -
 -
0.0 -
**
ATF6 (N) / Tubulin
**
**
**
0 3 6 12 24 36 48 (h)
*
**
 1.6 -
-
1.4 -
-
1.2 -
-
 1.0 -
-
0.8 -
-
0.6 -
-
0.4 -
-
0.2 -
 -
0.0 -
**
**
XBP1s / Tubulin
*
0 3 6 12 24 36 48 (h)
 1.6 -
-
1.4 -
-
1.2 -
-
 1.0 -
-
0.8 -
-
0.6 -
-
0.4 -
-
0.2 -
 -
0.0 -
cl-PARP / Tubulin
**
**
**
**
**
0 3 6 12 24 36 48 (h)
HT1376
 - + + + + TG (300 nM, 24 h)
0 0 10 20 40 si-XAF1 (pM)
P-IRE1α
IRE1α
XBP1s
cl-PARP
XAF1
Tubulin
100 -
100 -
55 -
90 -
45 -
35 -
45 -
IB
(KDa)
RT-PCR
(bp)
 XBP1u
 XBP1s
BLOC1S1
XAF1
GAPDH
283 -
257 -
461 -
237 -
387 -
B

## Slide 9
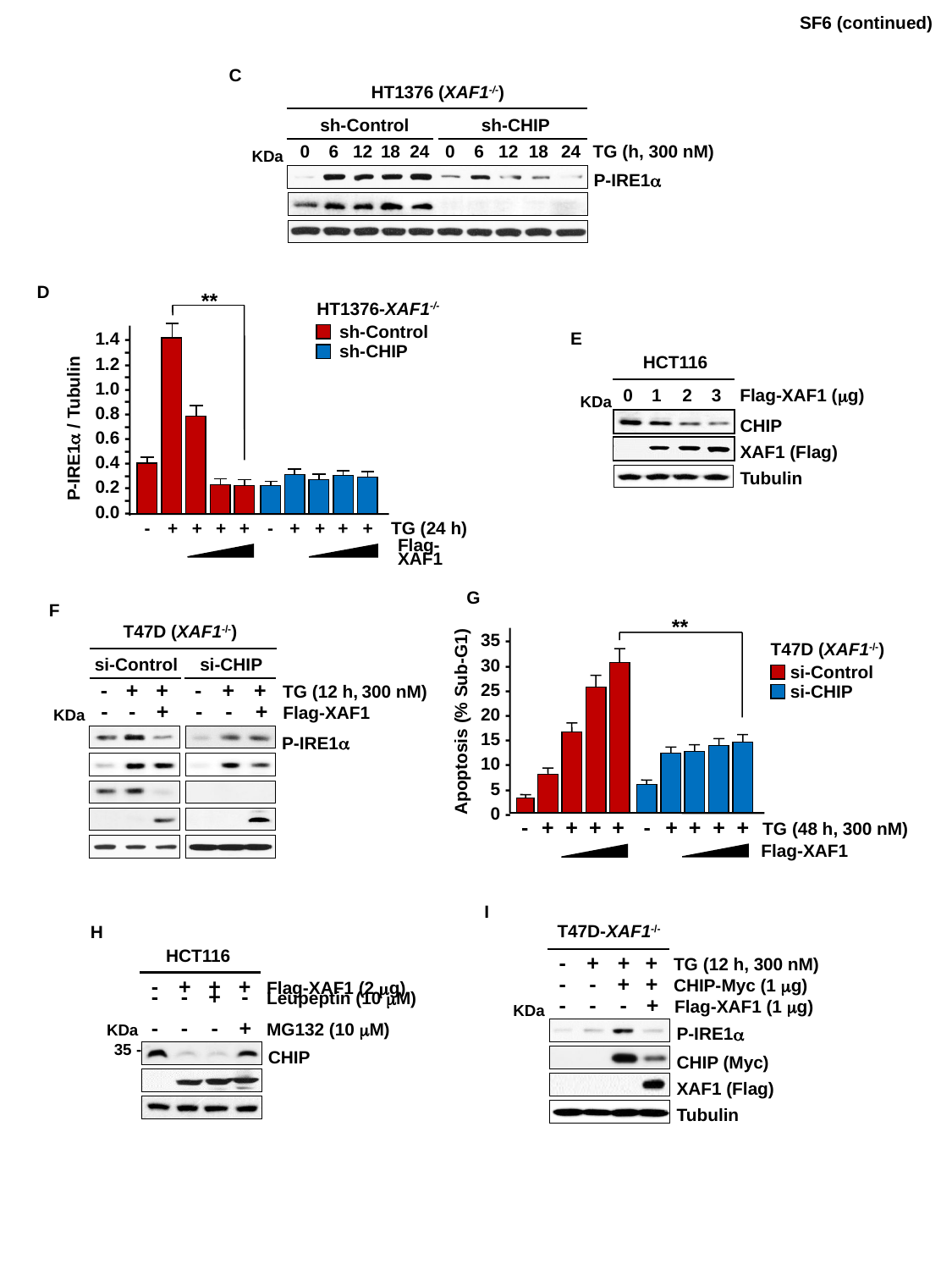

SF6 (continued)
C
HT1376 (XAF1-/-)
sh-Control sh-CHIP
0 6 12 18 24 0 6 12 18 24 TG (h, 300 nM)
KDa
P-IRE1α
CHIP
Tubulin
100 -
35 -
45 -
D
**
HT1376-XAF1-/-
sh-Control
sh-CHIP
 1.4 -
-
1.2 -
-
1.0 -
-
0.8 -
-
0.6 -
-
0.4 -
-
0.2 -
 -
0.0 -
P-IRE1α / Tubulin
Flag-XAF1
 - + + + + - + + + + TG (24 h)
E
HCT116
0 1 2 3 Flag-XAF1 (μg)
KDa
CHIP
XAF1 (Flag)
Tubulin
35 -
45 -
35 -
45 -
G
F
**
35 -
30 -
 25 -
20 -
 15 -
10 -
 5 -
0 -
T47D (XAF1-/-)
si-Control
si-CHIP
Apoptosis (% Sub-G1)
- + + + + - + + + + TG (48 h, 300 nM)
 Flag-XAF1
T47D (XAF1-/-)
si-Control si-CHIP
- + + - + + TG (12 h, 300 nM)
KDa
- - + - - + Flag-XAF1
P-IRE1α
IRE1α
CHIP
XAF1 (Flag)
Tubulin
100 -
100 -
35 -
45 -
35 -
45 -
I
T47D-XAF1-/-
- + + + TG (12 h, 300 nM)
- - + + CHIP-Myc (1 μg)
- - - + Flag-XAF1 (1 μg)
KDa
P-IRE1α
CHIP (Myc)
XAF1 (Flag)
Tubulin
100 -
35 -
45 -
35 -
45 -
H
HCT116
- + + + Flag-XAF1 (2 μg)
- - + - Leupeptin (10 μM)
KDa
- - - + MG132 (10 μM)
35 -
45 -
35 -
45 -
CHIP
XAF1 (Flag)
Tubulin
