# Supplementary material for: XAF1 drives apoptotic switch of endoplasmic reticulum stress response through destabilization of GRP78 and CHIP
Source: Cell Death Dis. 2022 Jul 28;13(7):655. doi: 10.1038/s41419-022-05112-0 (PMC9334361; doi:10.1038/s41419-022-05112-0)
Supplement: Supplementary file 2 — Supplementary information-Materials and Methods & SFigure legends [file 41419_2022_5112_MOESM2_ESM.docx]

**Materials and methods**

**Human cell lines and reagents**

Twenty human cancer cell lines derived from colon (RKO, LoVo, DLD-1, HCT116, SW620, SNU-C2A, SNU-C5, and LS174T), bladder (253J, T24, J82, and HT1376), and breast (HCC1937, MDA-MB231, BT-20, T47D, and Hs578T), and brain (A1207, LN18, and LN229) were purchased from the American Type Culture Collection (Rockville, MD, USA) or Korea Cell Line Bank (Seoul, Korea). Cell lines were maintained at 37°C in a 5% CO2 incubator and cultured in RPMI1640, McCoy, or DMEM medium supplemented with 10% fetal bovine serum (FBS) (GenDEPOT, Barker, TX, USA). Human haploid cell line HAP1 and its ZNF313 knockout subline were obtained from Horizon Discovery Ltd (Cambridge, UK). Cyclohexamide (CHX), Thapsigargin (TG), Tunicamycin (TM), Brefeldin A (BFA), GSK2606414, 4μ8C, AEBSF, 3‑MA, and Leupeptin were purchased from Sigma Aldrich (Saint Louis, MO, USA). BafA1 was purchased from Millipore Sigma. MG132 (HY‑13259) was purchased from AG Scientific Inc (CA, USA).

**XAF1-inducible and knockout cell lines**

Cell lines with tetracycline-inducible XAF1 system (Tet-XAF1) were generated by co-transfection of XAF1 (pcDNA4/TO) and tetracycline repressor vector (TetR) (Genolution Pharmaceuticals, Seoul, Korea) and selection under blasticidin and zeocin. The *XAF1* knockout sublines of J82, HT1376, T47D, and LoVo were generated using the CRISPR/Cas9 system. Cells were co-transfected with 1 μg of XAF1 CRISPR/Cas9 knockout plasmid (sc-402427, SantaCruz Biotechnology, CA, USA) and 2 μg of XAF1 HDR plasmid (sc-402427-HDR, SantaCruz Biotechnology). After 48 h, cells were screened with puromycin for 3 days. After the selection process, cells were seeded in the 96 well plates as single clones. Individual clones were screened for XAF1 expression using immunoblot assay and DNA-PCR. HT1376 *XAF1^-/-^* sublines with short hairpin (sh) RNA-mediated CHIP knockdown were established by transfection of sh-CHIP constructs (Genolution Pharmaceuticals Inc) and Zeocin (Invitrogen) selection.

**Expression plasmids and siRNAs**

Expression vectors for XAF1, GRP78, IRE1α, PERK, ATF6, ZNF313, ATF4, and NRF2 were constructed using a PCR-based approach. CHIP-Myc, HA-Ubiquitin-K63, and HA-Ubiquitin-K48 were purchased from Addgene. Xpress-tagged Ubiquitin plasmid was subcloned by inserting ubiquitin fragment of HA-Ub into His-Xpress vector (Invitrogen). Small interfering RNA (siRNA) duplexes against XAF1 (si-XAF1; 5ʹ-GAGCGCCCUGUUGAGUGUAAGUUCUGC-3ʹ), GRP78 (si-GRP78; 5ʹ-GGAGCGCAUUGAUACUAGA-3ʹ), ATF4 (si-ATF4; 5ʹ-CCAGAUCAUUCCUUUAGUUUA-3ʹ), ATF6 (si-ATF6; 5ʹ-GGUUAGAGGCGAGAUUAAA-3ʹ), CHIP (si-CHIP; 5ʹ -CUAUGAAGGAGGUUAUUGA-3ʹ), IRE1α (si-IRE1α; 5ʹ-GUAAAUUCAGGACCUAUAA-3ʹ), NRF2 (si-NRF2; 5ʹ-GGAUUAUUAUGACUGUUAA-3ʹ), PERK (si-PERK; 5ʹ-CAACCAUUGUGCUAAUAAA-3ʹ), XAF1 (si-XAF1; 5ʹ-GAGCGCCCUGUUGAGUGUAAGUUCUGC-3ʹ), and ZNF313 (siZNF313-1: 5ʹ-GCUGCCGUAAGAAUUUCUU-3ʹ) were synthesized by Genolution Inc (Seoul, Korea). Control siRNA duplex (AM4635) served as a negative control was purchased from Applied Biosystems (Thermo Fisher Scientiﬁc, Waltham, MA, USA). Transfection of siRNAs or expression plasmids was performed using Neon®Transfection System (Thermo Fisher Scientiﬁc) or E-fection in vitro Transfection Reagent (Lugen Sci co., Ltd, Bucheon, Korea).

**Reporter constructs and luciferase assay**

The *XAF1* promoter region was cloned into the pGL4.14 vector (Promega Corporation, Madison, USA). Cells were co-transfected with 200 ng of reporter plasmids and 20 ng of the β-galactosidase expression plasmid. β-galactosidase activity was measured by Mammalian β-Galactosidase Assay Kit (75707, Thermo Fisher Scientific) according to the manufacturer’s protocol and used for normalization. After normalization of each extract for protein content, luciferase activity was measured using Steady-Glo® Luciferase Assay System (E2510, Promega Corporation, Madison, USA) and SpectraMax i3x microplate reader (Molecular Devices, CA, USA). The assays were repeated three times, and the results were presented as mean values ± standard deviation (SD).

**Semi-quantitative reverse transcription-polymerase chain reaction (RT-PCR)**

Briefly, 1 μg of total cellular RNA was converted to cDNA by reverse transcription using random hexamer primers and MoMuLV reverse transcriptase (Invitrogen). PCR was initially performed over a range of cycles (20-40 cycles) by using serially diluted cDNA, and 1:4 diluted cDNA (12.5 ng per 50 μl of PCR) undergoing 24–38 cycles was found within the logarithmic phase of amplification with primers used for primers for *XAF1* (sense; 5ʹ-CAGAAGTCCTCGCTGGAGTTTC-3ʹ and antisense; 5ʹ-TGAAATTCTTTCCCCTTTCC-3ʹ), *GRP78* (sense; 5ʹ-GCTCGACTCGAATTCCAAAG-3ʹ and antisense; 5ʹ-TTTGTCAGGGGTCTTTCACC-3ʹ), *ATF6* (sense; 5ʹ-TCCGTGACTAAACCTGTCCTAC-3ʹ and antisense; 5ʹ-TGACAAGCGGATTCTCGATTTTT-3ʹ), *CHIP* (sense; 5ʹ-TCAAGGAGCAGGGCAATCGTCT-3ʹ and antisense; 5ʹ-GCATCTTCAGGTAGCACAAGGC-3ʹ), *IRE1α* (sense; 5ʹ-GA GACGTCATTGCACGTGAATT-3ʹ and antisense; 5ʹ-AGGTCCTGAATTTACGCAGGT-3ʹ), NRF2 (sense; 5ʹ-GAGAGCCCAGTCTTCATTGC-3ʹ and antisense; 5ʹ-TGCTCAATGTCCTGTTGCAT-3ʹ), *PERK* (sense; 5ʹ-ATC CCCCATGGAACGACCTG-3ʹ and antisense; 5ʹ-ACCCGCCAGGGACAAAAATG-3ʹ), *NRF2* (sense; 5ʹ-GAGAGCCCAGTCTTCATTGC-3ʹ and antisense; 5ʹ-TGCTCAATGTCCTGTTGCAT-3ʹ), *XBP1* (sense; 5ʹ-TTACGAGAGAAAACTCATGGCC-3ʹ and antisense; 5ʹ-GGGTCCAAGTTGTCCAGAATGC-3ʹ), *BLOC1S1* (sense; 5ʹ- GAGGCGAGAGGCTATCACTG-3ʹ and antisense; 5ʹ- GGAGGCTTGTGTTTTATTCAAGG-3ʹ), and an endogenous expression standard gene *GAPDH*. PCR products were resolved on 2% (wt/vol) agarose gels and visualized on a GelDoc (BioRad, Hercules, CA, USA). Quantitation was achieved by densitometric scanning of the ethidium bromide-stained gels. Integration and analysis was performed by using Quantity One software program (Bio-Rad). The assays were repeated at least three times, and the results were presented as mean values ± standard deviation (SD).

**Chromatin immunoprecipitation (ChIP)**

ChIP assay was carried out using a Simple ChIP™ Enzymatic Chromatin IP Kit (#9003, Cell Signaling Technology, Danvers, MA, USA). Briefly, cells were fixed by 1% formaldehyde for 5 min and then sonicated to prepare chromatin suspensions. Immunoprecipitation analysis was carried out with antibodies specific for NRF2 (#12721, Cell Signaling Technology) or normal rabbit IgG (#2729, Cell Signaling Technology). The *XAF1* promoter region comprising the putative NRF2-binding site (ARE) was amplified using primers P3 (sense; 5ʹ-CCAGCTCTGATGTTGAGCGA-3ʹ) and RP1 (antisense; 5ʹ-TTGCTATGGAAAACAGAGGCAGT-3ʹ).

**Immunoblot and immunoprecipitation**

Antibodies specific for ATF4 (#11815), ATF6 (ab122987), β-tubulin (T-0198), CHIP (#2895), CHOP (#2895), cleaved PARP (#9541), cleaved Caspase-3 (#9665), eIF2α (#5324), Phospho-eIF2α (#3398), FLAG (sc-1666384), GFP (sc-9996), GRP78/BiP (ab21685, #3177, sc-13539), GST (sc-33613), HA (Y-11; sc-805), LC3 (#4108), IRE1α (#3294), Phospho-IRE1α (ab48187), PERK (#5683), Phospho-PERK (ab192591), NRF2 (A-10; sc365949, #12721), Ub (sc-8017), XBP-1s (#12782), XAF1 (sc-398012, sc-374020, #13805) and ZNF313 (sc-101116) were purchased from Santa Cruz Biotechnology (Santa Cruz, CA, USA), Cell Signaling Technology, Abcam (Cambridge, MA, USA), and Sigma Aldrich. Antibody binding was detected by enhanced chemiluminescence (Amersham Biosciences) using a secondary antibody conjugated to horseradish peroxidase.Quantitation of immunoblots was achieved by densitometric scanning of the bands. Integration and analysis was performed by using Quantity One software program (Bio-Rad). The assays were repeated three times, and the results were presented as mean values ± standard deviation (SD).

**Immunohistochemistry**

Immunohistochemistry assay for human breast tissues was performed using tissue array slides (US Biomax, MD, USA) and Vectastain ABC (avidin–biotin–peroxidase) kit (Vector Laboratories). Briefly, slides were incubated with XAF1 or GRP78 antibody overnight at 4°C and visualized using an Axio Scan.Z1 microscopy (Carl Zeiss MicroImaging, Inc). The expression levels of XAF1 and GRP78 were evaluated using Zen software (Zeiss, Oberkochen, Germany). For the immunoreactive score, we established as 1 to 5 point system by multiplying the percentage of positive cells by the intensity of the staining score.

**Immunofluorescence and microscopic autophagy assay**

Cells were fixed with 4% formaldehyde, permeabilized with 100% methanol, and blocked 0.1% Triton X-100. The cells were incubated with anti-XAF1 (A-11, Santa Cruz) antibody at 4°C overnight. Next, cells were washed and incubated for 1 h at room temperature with Alexa488-conjugated anti-rabbit (1:250, A21206), Alexa594-conjugated anti-mouse (1:250, A21206), or Alexa488-conjugated anti-mouse (1:250, A21202) secondary antibodies (Thermo Fisher Scientific). The fluorescence and DIC images were detected using a confocal laser scanning microscope (LSM-800, Carl Zeiss MicroImaging Inc). For autophagy assay, T47D cells were seeded in 2‑well chamber slide (154461, Thermo Fisher Scientific) and co‑transfected with GFP‑LC3B and either red fluorescent protein (RFP)-control or RFP-XAF1. The cells were treated with TG (200 nM) for 48 h. 3-MA (5 μM) or BafA1 (100 nM) were added 2 h before TG treatment. The cells were fixed in 4% paraformaldehyde for 10 min and fluorescent images of cells showing punctuated changes of GFP‑LC3 were obtained with the confocal microscope (LSM510META, Carl Zeiss AG). A minimum of 150 transfected cells were counted to quantify the puncta signals for each sample.

**Protein pull-down and *in vitro* binding assay**

For *in vitro* binding assay, GST-fused XAF1 (GST-XAF1) proteins overexpressed by IPTG in BL21 strain were purified using Glutathione Sepharose 4B (GE Healthcare, Little Chalfont, UK). N-terminal His-tagged recombinant human ZNF313 and recombinant human GRP78 (ab79139) were purchased from Abcam. The GST-XAF1, r-GRP78 and r-His-ZNF313 proteins were incubated with binding assay buffer for 6 h. Immunocomplexes were separated by incubation with protein-A/G Sepharose for 1 h and subjected to SDS-PAGE for immunoblot analysis.

**Ubiquitination assay**

Cells were transfected with HA-K63 Ub, HA-K48 Ub or Xpress-Ub plasmids, and expression vectors. Cells were incubated with MG132 (20 μM) for 6 h and cell extracts were prepared in buffer containing complete protease inhibitor (Roche). The lysates were incubated with GRP78 antibody overnight at 4 °C, and protein complexes were pelleted with protein A-agarose beads (Thermo Fisher Scientific). The beads were then washed three times with washing buffer (1M NaCl, 10 mM Tris-Cl (pH 8), 1 mM EDTA, 1% NP-40), and ubiquitin conjugated proteins were eluted by boiling in 2X protein sample buffer and visualized by immunoblot assay.

**Apoptosis assay**

For flow cytometry assay, cells (5 x 10^4^) were seeded in six‑well plate in triplicate and transfected with expression vector or siRNA. For sub-G1 fraction analysis, cells were fixed with 70% ethanol and resuspended in 1 ml of PBS containing 100 mg/ml RNase and 50 mg/ml propidium iodide (Sigma). The assay was performed on a FACS Calibur flow cytometer (BD Bioscience) and the cell profile was analyzed using MultiCycle software (Phoenix Flow Systems).

**Animal studies**

Six-week‑old immunodeficient male nude mice (nu/nu) (Orient Bio Inc., Seongnam, Korea) were maintained in pressurized ventilated cages. Briefly, the identical numbers (1 x 10^6^) of LoVo subline cells were injected subcutaneously to three mice per group. Animal studies were repeated twice. Tumor growth was monitored periodically, and volume (V) was calculated by using the modified ellipsoidal formula: V = 1/2 × length × (width)^2^. At day 17, 3 mice of each group were exposed to normal saline or TG (12 μg/kg) by intratumoral injection, and tumor volume was measured at the beginning of injection and monitored regularly for 29 days. All studies were performed with the approval of Korea University Institutional Animal Care and Use Committee and Korea Animal Protection Law.

**Statistical analysis**

All experiments, including RT-PCR, reporter luciferase and flow cytometry assays were repeated three times, and the results were presented as mean values ± standard deviation (SD). Mean and SD values were calculated using Microsoft Excel software. A student’s *t*‑test (GraphPad Prism8 software, CA, USA) was performed to determine the statistical significance. A *P* value of less than 0.05 was considered significant.

**Legends for Supplementary Figures**

**Fig. S1** **A** Recovery of apoptotic sensitivity of J82-*XAF1^-/-^* subline cells by ectopic expression of XAF1. Cells were transfected with 2 μg of pcDNA (control) or Flag-XAF1 plasmids. IB assay of cleaved PARP and cl-CASP3 levels was carried out to detect TG-induced apoptosis. **B** Apoptosis-promoting effect of XAF1. T47D cells were transfected with pcDNA (control) or Flag-XAF1 plasmids and then exposed to an increasing dose of TG as indicated. Apoptosis induction was measured by flow cytometric analysis of sub-G1 fraction. **C** Effect of XAF1 depletion and expression on TG-induced apoptosis. Cells were transfected with an increasing dose of si-XAF1 or Flag-XAF1 and then exposed to TG (300 nM, 48 h). **D-F** XAF1 stimulation of TG-induced autophagy and effect of its inhibition on apoptosis induction. J82 and T47D sublines were exposed to 3‑MA (5 μM) at 2 h before TG treatment (200 nM). **G** Autophagy-promoting effect of XAF1. *XAF1^+/+^* and *XAF1^-/-^* sublines of LoVo were treated with TG as indicated. LC3-I/II, Beclin-1, and Atg5-Atg12 were used as markers of autophagy induction. **H** Effect of XAF1 depletion on TG-induced autophagy and apoptosis in LoVo cells. **I** Effect of XAF1 induction on TG-induced autophagy and apoptosis in T47D cells. XAF1 induction was achieved by Tet-inducible XAF1 (Tet-XAF1) system. **J, K** Immunofluorescence microscopic analysis of LC3B puncta showing autophagy-stimulating function of XAF1. GFP-LC3 and RFP-XAF1 were transfected to detect LC3B puncta and XAF1 expression, respectively. Cells were exposed to 3‑MA (5 μM) or BafA1 (100 nM) at 2 h before TG treatment (200 nM). Data represent the mean ± SD of triplicate assays. * *P* < 0.05; ** *P* < 0.01 (Student *t* test).

**Fig. S2** **A** XAF1 activation of TG-mediated UPR signaling. Cells were transfected with an increasing dose of Flag-XAF1. The cells were treated with TG for 6 h. **B** Effect of XAF1 knockdown on TG activation of UPR signaling factors in LoVo cells. **C** Quantitative analysis of expression levels of GRP78 (Fig. 4B). Quantitation was achieved by densitometric scanning of the band intensities on immunoblots. Data represent the mean ± SD of triplicate assays. ** *P* < 0.01 (Student *t* test). **D** Upregulation of GRP78 protein level by XAF1 depletion. HCC1937 cells were transfected with an increasing dose of si-XAF1. IB assay was carried out at 48 h after transfection.

**Fig. S3 A** Immunofluorescence microscopic analysis of cellular localization of GRP78 and XAF1 in HT1376 cells. DAPI was used for counterstaining of the nuclei. **B** No apoptosis-promoting activity of Δ7C-XAF1. Cells were transfected with either WT-XAF1 or Δ7C-XAF1 and then treated with TG (300 nM). Apoptosis induction was determined by flow cytometric analysis of sub-G1 fraction. Data represent the mean ± SD of triplicate assays. ** *P* < 0.01 (Student *t* test).

**Fig. S4** **A** Quantitative analysis of expression levels of GRP78 (Fig. 4A). Quantitation was achieved by densitometric scanning of the band intensities on immunoblots. Data represent the mean ± SD of triplicate assays. ** *P* < 0.01 (Student *t* test). **B** Reduction of GRP78 protein level by ZNF313 expression. HCT116 cells were transfected with an increasing dose of V5-ZNF313. IB assay was carried out at 48 h after transfection. **C** IP assay showing ZNF313 induction of GRP78 ubiquitination. **D** Immunofluorescence microscopic analysis of cellular localization of GRP78 and ZNF313 in HT1376 cells. DAPI was used for counterstaining of the nuclei. **E** Effect of ZNF313 depletion on TG-induced apoptosis. HCT116 cells were transfected with 50 pM of si-Control or si-ZNF313 and then exposed to TG (300 nM) for 6, 12, and 24 h. Apoptosis induction was determined by immunoblot assay of cleaved PARP level and flow cytometric analysis of sub-G1 fraction. Data represent the mean ± SD of triplicate assays. ** *P* < 0.01 (Student *t* test).

**Fig. S5 A** XAF1 induction by TG and its association with GRP78 expression and apoptosis induction in human cell lines. **B** Induction of *XAF1* mRNA in response to ER stress inducers. Cells were treated with TG, TM, and BFA for 12 h. **C** Blockade of TG induction of *XAF1* mRNA by actinomycin D (Act D). Cells were pretreated with Act D for 2 h before TG exposure. **D** Activation of *XAF1* mRNA expression by cytotoxic doses of TG in HCC1937 cells. **E** Loss of cytotoxic TG effect by blockade of XAF1 induction. **F, G** Comparison of GRP78 induction kinetics between J82-*XAF1^+/+^* and J82-*XAF1^-/-^* subline cells. Cells were treated with TG (300 nM) and TM (1 μg/ml) as indicated. **H** IP assay showing the interaction of upregulated XAF1 and GRP78 proteins in J82 cells exposed to TG (300 nM). **I** XAF1 blockade effect on TG-induced GRP78 expression. HCT116 cells were transfected with si-XAF1 as indicated and then exposed to TG (300 nM, 12 h). **J, K** Disruption of TG induction of *XAF1* by depletion of PERK or pretreatment of GSK2606414.**L** Disruption of TG induction of *XAF1* by Nrf2 depletion. **M** XAF1 induction by ectopic expression of PERK and its attenuation by Nrf2 depletion. **N** TG activation of the reporter containing the putative ARE (Pro1123-Luc) and its attenuation by Nrf2 depletion. Cells were transfected with the reporter and then exposed to TG (300 nM) for 12 h.

**Fig. S6** **A** Quantitative analysis of expression levels of GRP78 and UPR transducers (Fig. 6A). Quantitation was achieved by densitometric scanning of the band intensities on immunoblots.

Data represent the mean ± SD of triplicate assays. ** *P* < 0.01 (Student *t* test). **B** Effect of XAF1 depletion on TG-induced phosphorylation of IRE1α and expression of *XBP1u/XBP1s* and *BLOC1S1* mRNA. HT1376 cells were transfected with an increasing dose of si-XAF1 and then treated with TG (300 nM) for 24 h. RT-PCR was performed to determine expression levels of spliced form of *XBP1* (*XBP1s*) and full-length of *BLOC1S1*. *XBP1u*, unspliced *XBP1*. **C** Effect of CHIP knockdown on TG-induced IRE1α phosphorylation. IRE1α phosphorylation was compared between sh-Control and sh-CHIP sublines of HT1376-*XAF1^-/-^* cells. **D** Quantitative analysis of GRP78 expression (Fig. 6E). Quantitation was achieved by densitometric scanning of the band intensities on immunoblots. Data represent the mean ± SD of triplicate assays. ** *P* < 0.01 (Student *t* test). **E** Effect of XAF1 overexpression on CHIP protein level. HCT116 cells were transfected with an increasing dose of Flag-XAF1 and IB assay was performed at 48 h after transfection. **F, G** IB and flow cytometric assays showing the CHIP dependency of XAF1 inhibition of IREα phosphorylation and XAF1 promotion of TG-mediated apoptosis. T47D-*XAF1^-/-^* cells were co-transfected with si-CHIP (50 pM) and Flag-XAF1 (2 μg) as indicated and then exposed to TG (300 nM) for 12 h. **H** Blockade of XAF1-induced CHIP degradation by MG132. HCT116 cells transfected with Flag-XAF1 were exposed to MG132 (10 μM) or Leupeptin (10 μM) for 6 h before harvest. **K** XAF1 suppression of CHIP-mediated IRE1α phosphorylation. T47D-*XAF1^-/-^* cells were transfected with Flag-XAF1 and/or CHIP-Myc as indicated and then exposed to TG (300 nM) for 12 h.
