# Supplementary material for: XAF1 drives apoptotic switch of endoplasmic reticulum stress response through destabilization of GRP78 and CHIP
Source: Cell Death Dis. 2022 Jul 28;13(7):655. doi: 10.1038/s41419-022-05112-0 (PMC9334361; doi:10.1038/s41419-022-05112-0)
Supplement: Supplementary file 4 — Western blots - Uncropped [file 41419_2022_5112_MOESM4_ESM.pptx]

## Slide 1
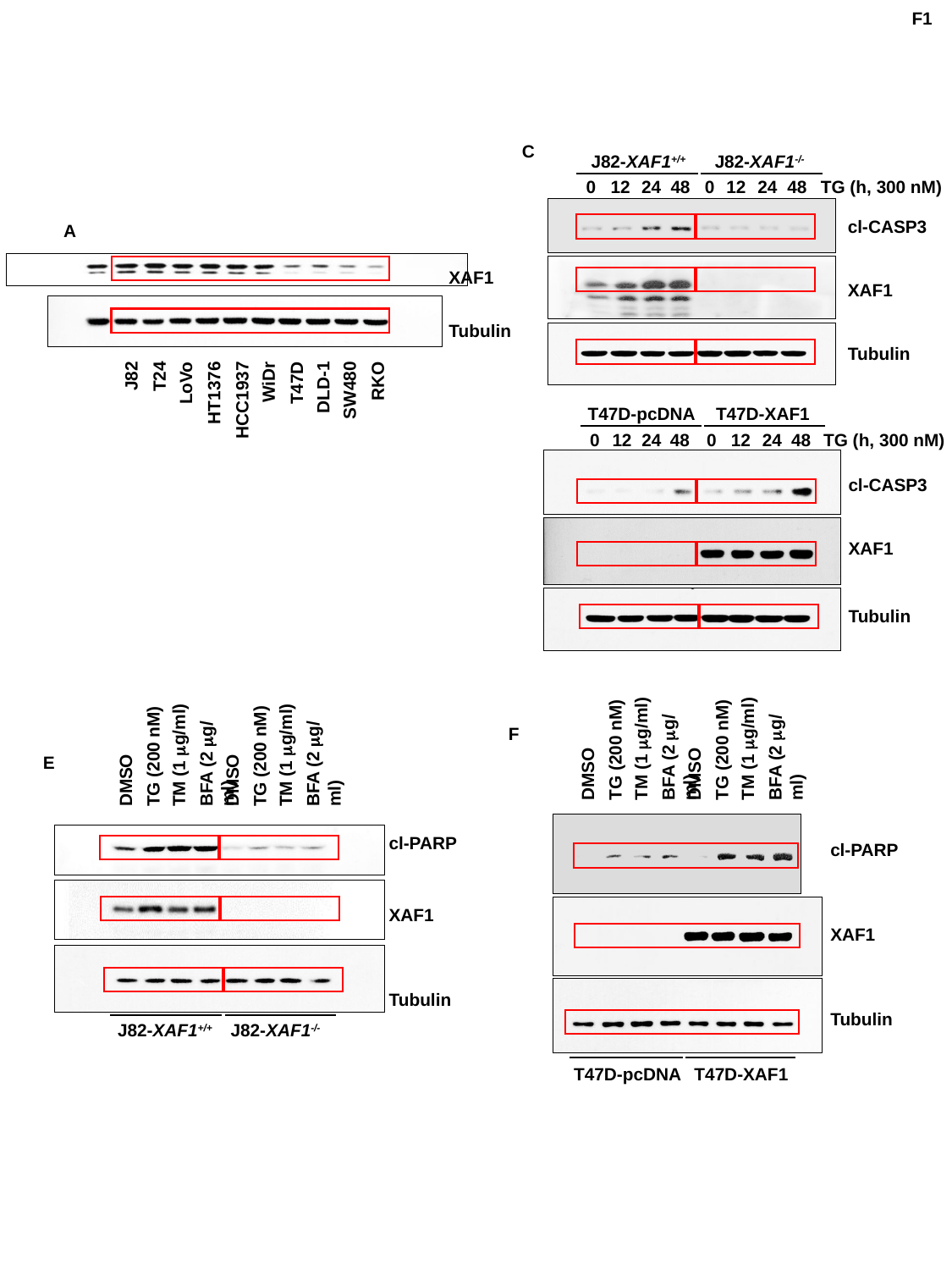

F1
C
J82-XAF1+/+ J82-XAF1-/-
0 12 24 48 0 12 24 48 TG (h, 300 nM)
cl-CASP3
XAF1
Tubulin
T47D-pcDNA T47D-XAF1
0 12 24 48 0 12 24 48 TG (h, 300 nM)
cl-CASP3
XAF1
Tubulin
A
XAF1
Tubulin
J82
T24
LoVo
HT1376
HCC1937
WiDr
T47D
DLD-1
SW480
RKO
DMSO
TG (200 nM)
TM (1 μg/ml)
BFA (2 μg/ml)
DMSO
TG (200 nM)
TM (1 μg/ml)
BFA (2 μg/ml)
F
cl-PARP
XAF1
Tubulin
T47D-pcDNA T47D-XAF1
DMSO
TG (200 nM)
TM (1 μg/ml)
BFA (2 μg/ml)
DMSO
TG (200 nM)
TM (1 μg/ml)
BFA (2 μg/ml)
E
cl-PARP
XAF1
Tubulin
J82-XAF1+/+ J82-XAF1-/-

## Slide 2
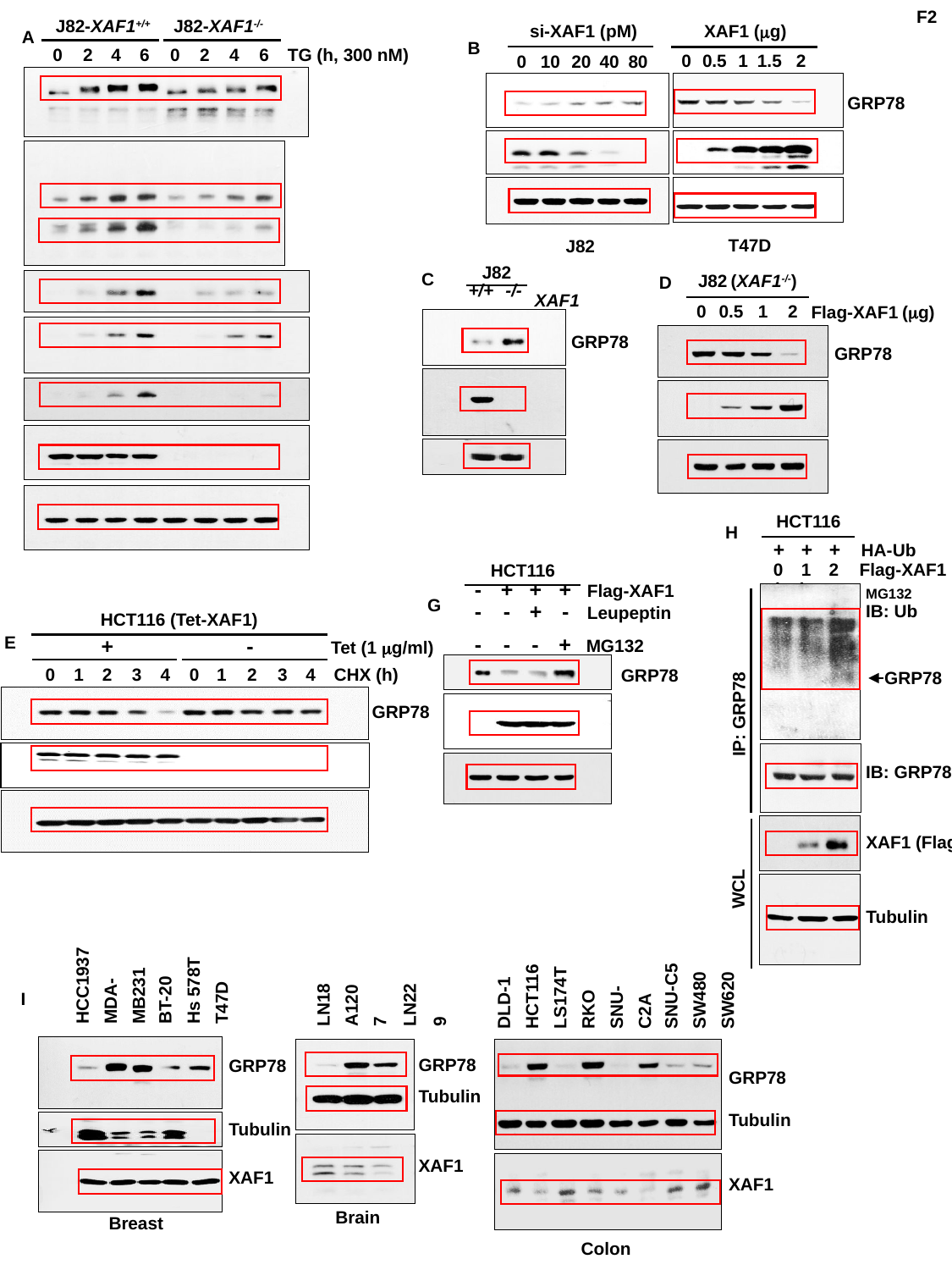

F2
J82-XAF1+/+ J82-XAF1-/-
A
0 2 4 6 0 2 4 6 TG (h, 300 nM)
PERK
P-IRE1α
CHOP
ATF6 (N)
ATF4
XBP1s
XAF1
Tubulin
XAF1 (μg)
 si-XAF1 (pM)
B
0 0.5 1 1.5 2
0 10 20 40 80
GRP78
XAF1
Tubulin
T47D
J82
J82
C
+/+ -/-
XAF1
GRP78
XAF1
Tubulin
J82 (XAF1-/-)
D
0 0.5 1 2
Flag-XAF1 (μg)
GRP78
XAF1 (Flag)
Tubulin
HCT116
H
+ + + HA-Ub
0 1 2 Flag-XAF1 (μg)
MG132
IB: Ub
IB: GRP78
GRP78
IP: GRP78
XAF1 (Flag)
Tubulin
WCL
HCT116
- + + + Flag-XAF1
G
- - + - Leupeptin
- - - + MG132
GRP78
XAF1 (Flag)
Tubulin
HCT116 (Tet-XAF1)
E
+ - Tet (1 μg/ml)
0 1 2 3 4 0 1 2 3 4 CHX (h)
GRP78
XAF1
Tubulin
HCC1937
MDA-MB231
BT-20
Hs 578T
T47D
DLD-1
HCT116
LS174T
RKO
SNU-C2A
SNU-C5
SW480
SW620
LN18
A1207
LN229
I
GRP78
Tubulin
XAF1
GRP78
Tubulin
XAF1
GRP78
Tubulin
XAF1
Brain
Breast
Colon

## Slide 3
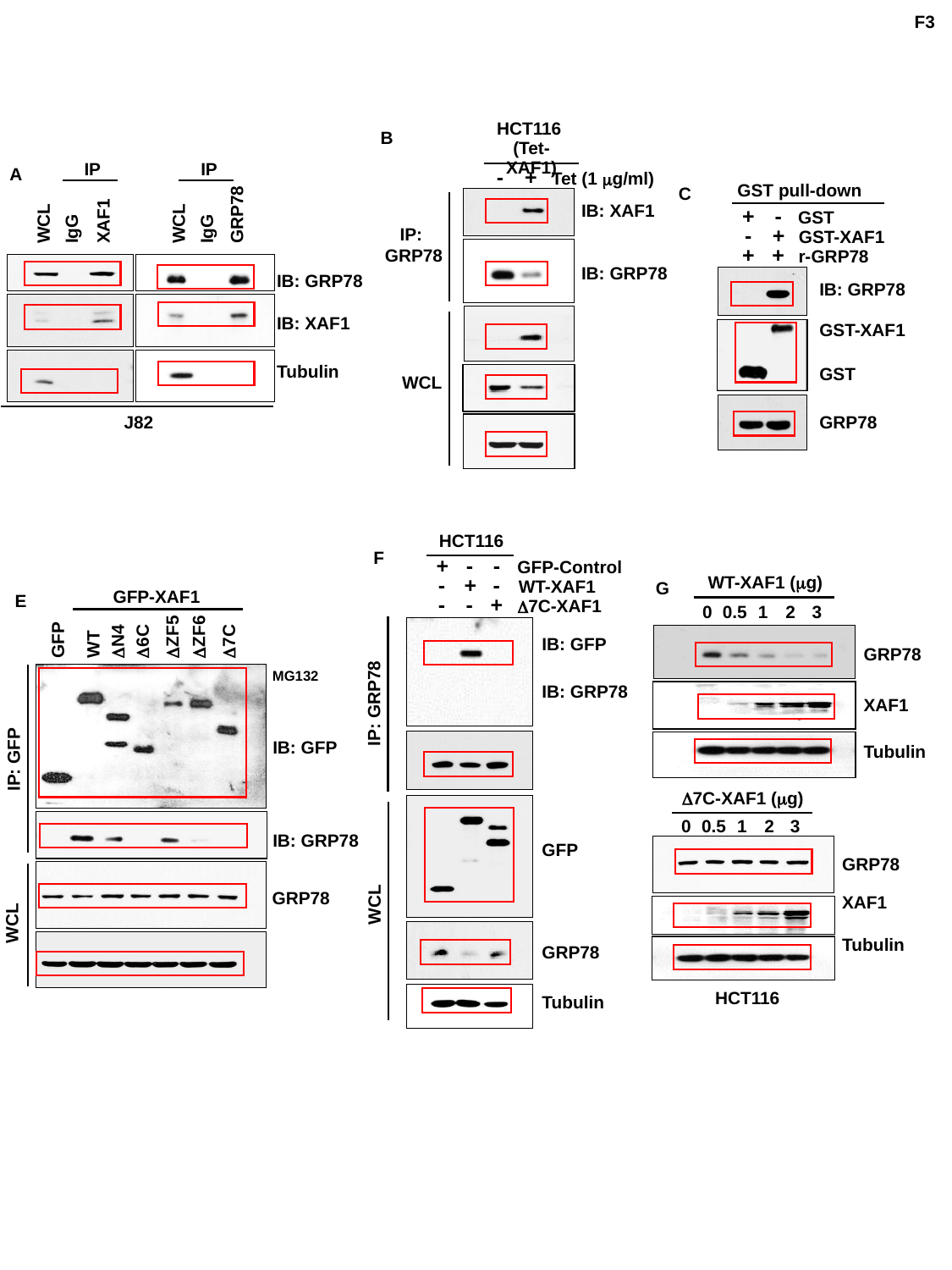

F3
HCT116
(Tet-XAF1)
B
- + Tet (1 μg/ml)
IB: XAF1
IB: GRP78
XAF1
GRP78
Tubulin
IP:
GRP78
WCL
IP
IP
A
WCL
IgG
GRP78
WCL
IgG
XAF1
IB: GRP78
IB: XAF1
Tubulin
J82
GST pull-down
C
+ - GST
 - + GST-XAF1
+ + r-GRP78
IB: GRP78
GST-XAF1
GST
GRP78
HCT116
F
+ - - GFP-Control
 - + - WT-XAF1
 - - + Δ7C-XAF1
IB: GFP
IB: GRP78
GFP
GRP78
Tubulin
IP: GRP78
WCL
WT-XAF1 (μg)
G
0 0.5 1 2 3
GRP78
XAF1
Tubulin
Δ7C-XAF1 (μg)
0 0.5 1 2 3
GRP78
XAF1
Tubulin
HCT116
GFP
WT
ΔN4
Δ6C
ΔZF5
ΔZF6
Δ7C
GFP-XAF1
E
MG132
IB: GFP
IB: GRP78
IP: GFP
GRP78
Tubulin
WCL

## Slide 4
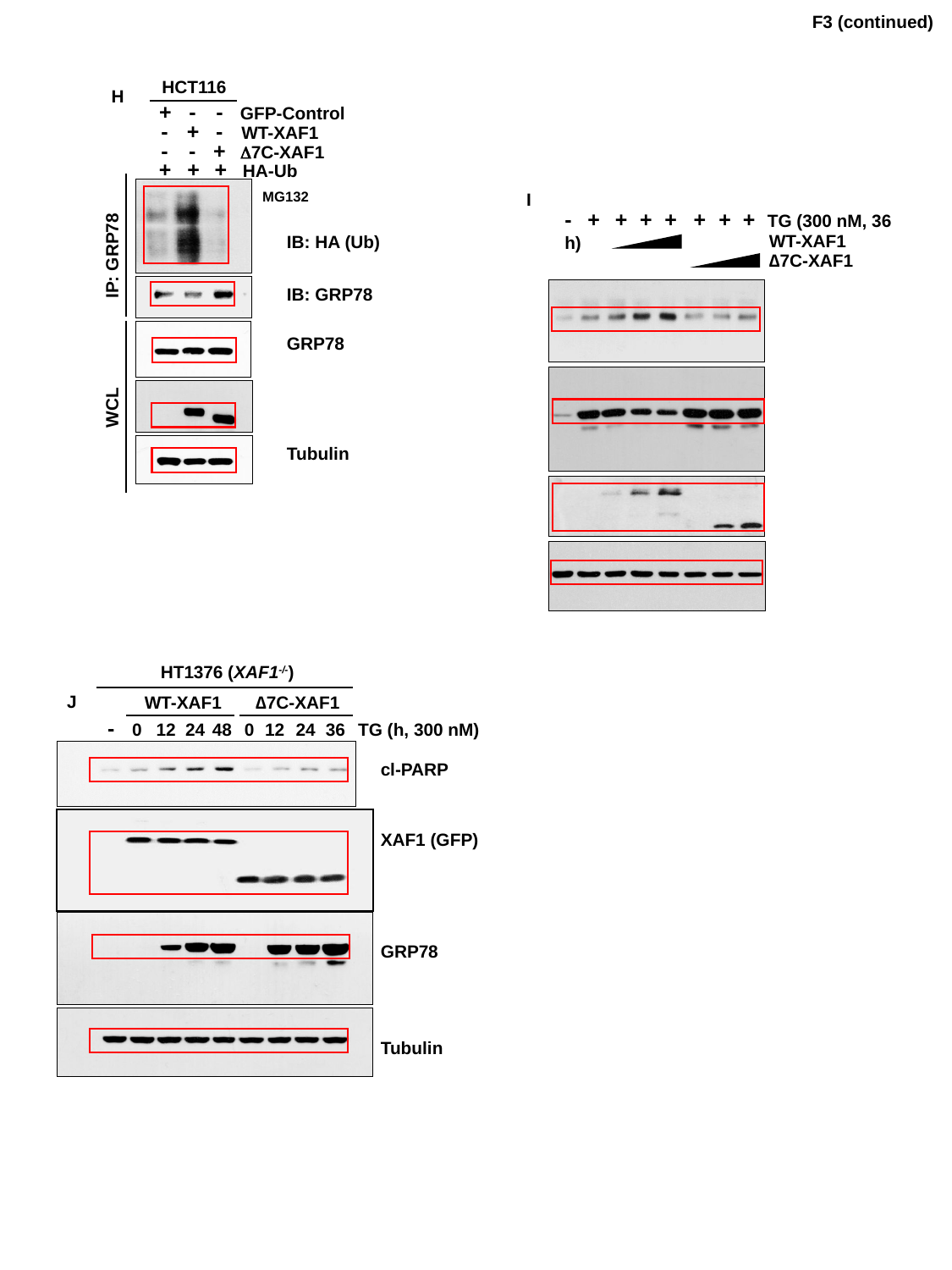

F3 (continued)
HCT116
H
+ - - GFP-Control
 - + - WT-XAF1
 - - + Δ7C-XAF1
+ + + HA-Ub
MG132
IB: HA (Ub)
IB: GRP78
GRP78
XAF1 (GFP)
Tubulin
IP: GRP78
WCL
I
- + + + + + + + TG (300 nM, 36 h)
WT-XAF1
∆7C-XAF1
cl-PARP
GRP78
XAF1
Tubulin
HT1376 (XAF1-/-)
J
WT-XAF1 ∆7C-XAF1
- 0 12 24 48 0 12 24 36 TG (h, 300 nM)
cl-PARP
XAF1 (GFP)
GRP78
Tubulin

## Slide 5
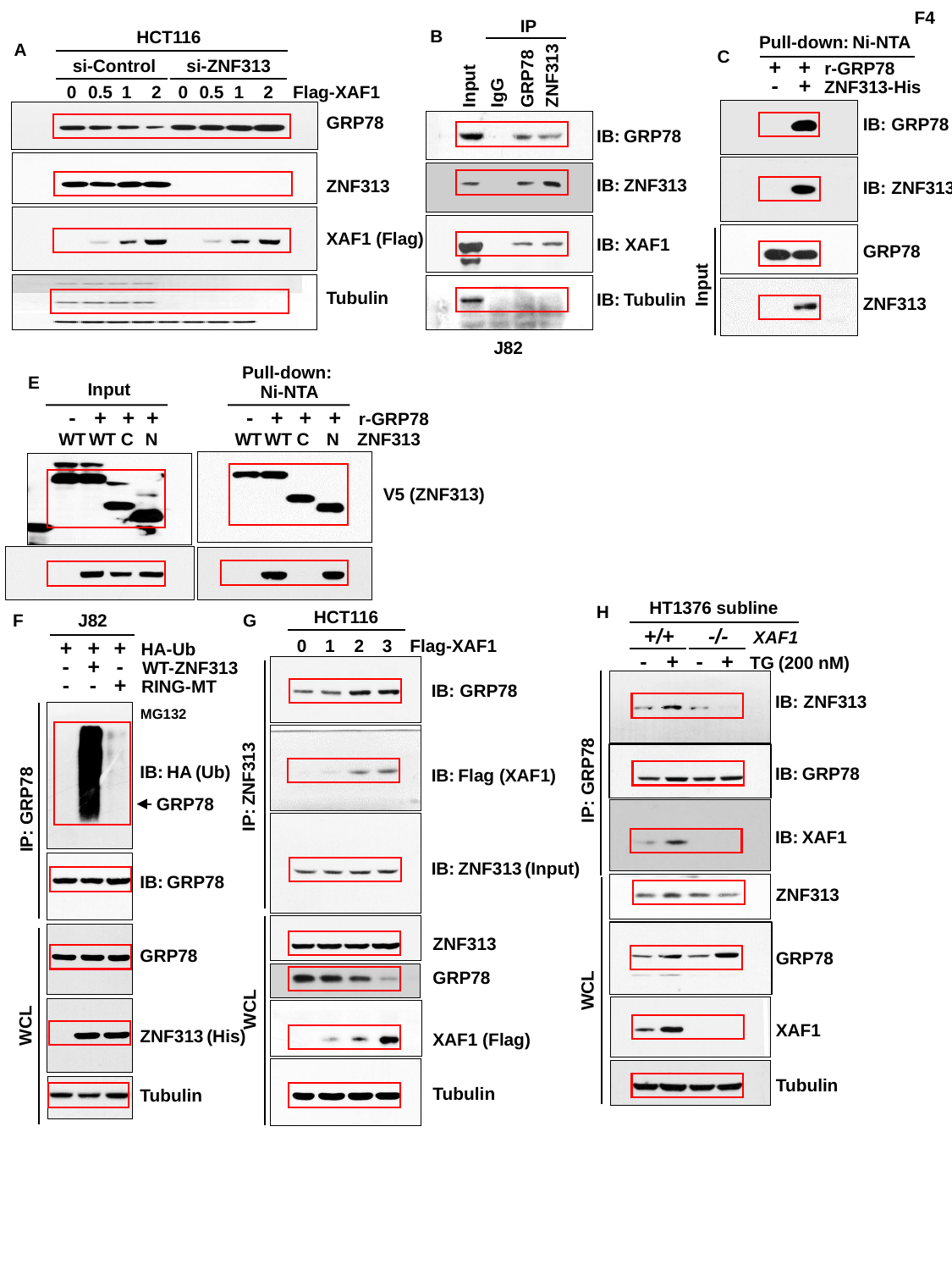

F4
IP
Input
IgG
GRP78
ZNF313
B
IB: GRP78
IB: ZNF313
IB: XAF1
IB: Tubulin
J82
HCT116
A
si-Control si-ZNF313
0 0.5 1 2 0 0.5 1 2 Flag-XAF1 (μg)
GRP78
ZNF313
XAF1 (Flag)
Tubulin
Pull-down: Ni-NTA
C
+ + r-GRP78
 - + ZNF313-His
IB: GRP78
IB: ZNF313
GRP78
ZNF313
Input
Pull-down:
Ni-NTA
E
Input
 - + + + - + + + r-GRP78
WT WT C N WT WT C N ZNF313
V5 (ZNF313)
GRP78
HT1376 subline
H
+/+ -/- XAF1
- + - + TG (200 nM)
IB: ZNF313
IB: GRP78
IB: XAF1
IP: GRP78
ZNF313
GRP78
XAF1
Tubulin
WCL
HCT116
G
0 1 2 3 Flag-XAF1 (μg)
IB: GRP78
IB: Flag (XAF1)
IB: ZNF313 (Input)
IP: ZNF313
ZNF313
GRP78
XAF1 (Flag)
Tubulin
WCL
F
J82
+ + + HA-Ub
- + - WT-ZNF313
- - + RING-MT
MG132
IB: HA (Ub)
IB: GRP78
GRP78
 IP: GRP78
GRP78
ZNF313 (His)
Tubulin
WCL

## Slide 6
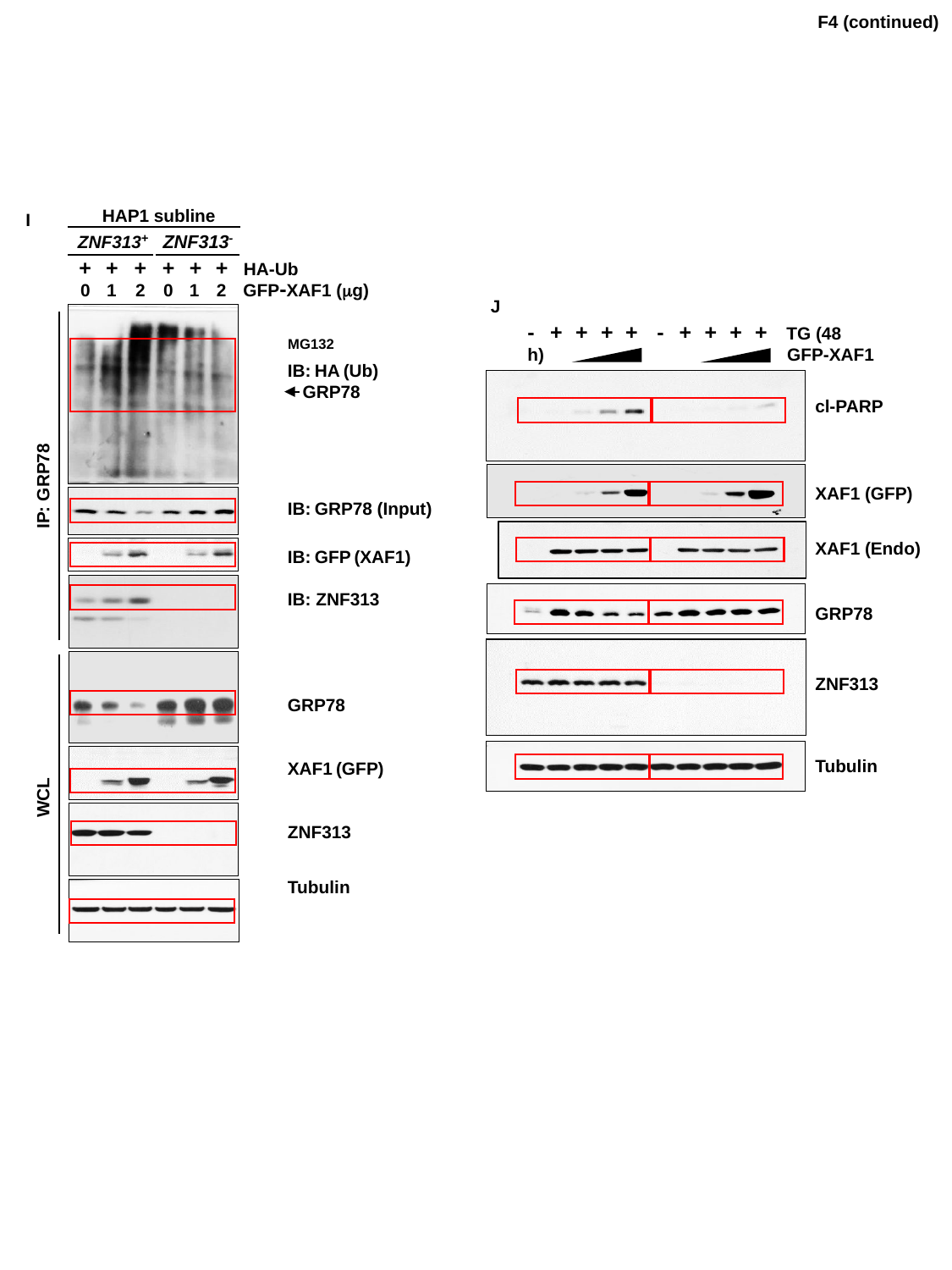

F4 (continued)
HAP1 subline
I
ZNF313+ ZNF313-
+ + + + + + HA-Ub
 0 1 2 0 1 2 GFP-XAF1 (μg)
MG132
IB: HA (Ub)
IB: GRP78 (Input)
GRP78
 IP: GRP78
IB: GFP (XAF1)
IB: ZNF313
GRP78
XAF1 (GFP)
ZNF313
Tubulin
WCL
J
- + + + + - + + + + TG (48 h)
GFP-XAF1
cl-PARP
XAF1 (GFP)
XAF1 (Endo)
GRP78
ZNF313
Tubulin

## Slide 7
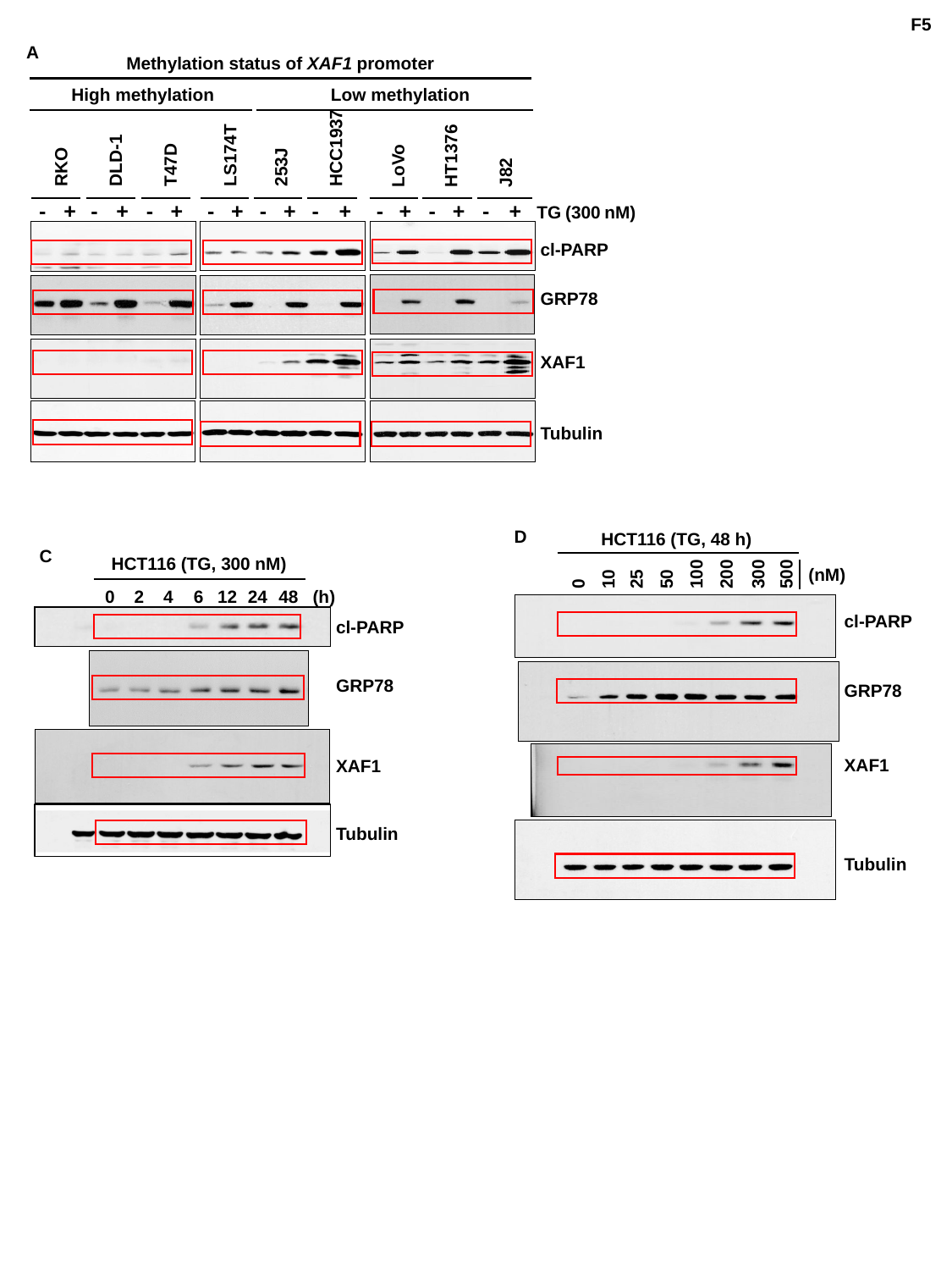

F5
A
Methylation status of XAF1 promoter
High methylation Low methylation
LS174T
253J
HCC1937
LoVo
HT1376
J82
RKO
DLD-1
T47D
- + - + - + - + - + - + - + - + - + TG (300 nM)
cl-PARP
GRP78
XAF1
Tubulin
0
10
25
50
100
200
300
500
D
HCT116 (TG, 48 h)
(nM)
cl-PARP
GRP78
XAF1
Tubulin
C
HCT116 (TG, 300 nM)
0 2 4 6 12 24 48 (h)
cl-PARP
GRP78
XAF1
Tubulin

## Slide 8
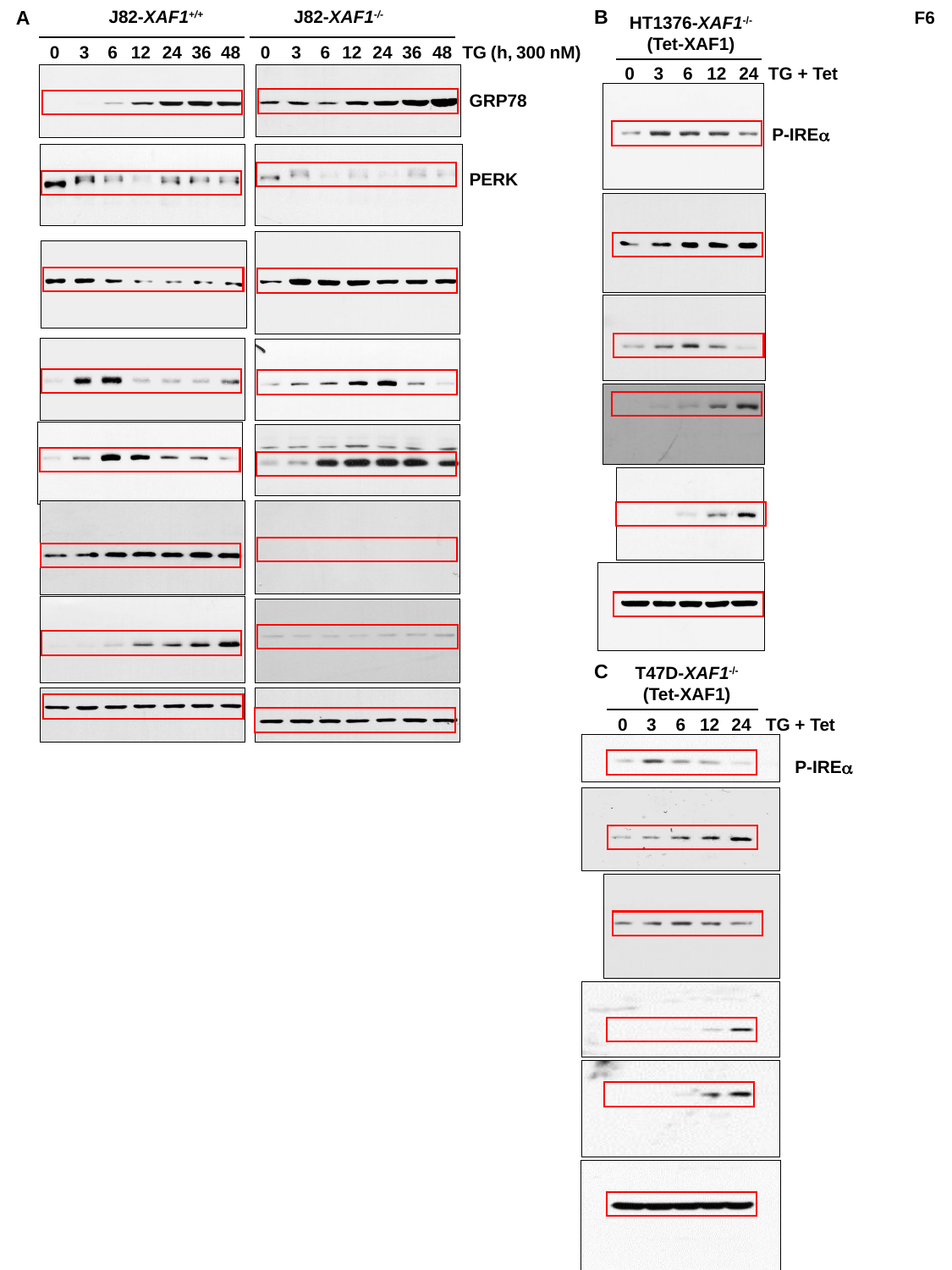

B
HT1376-XAF1-/-
(Tet-XAF1)
0 3 6 12 24 TG + Tet
P-IREα
IRE1α
XBP1s
cl-PARP
XAF1
Tubulin
J82-XAF1+/+ J82-XAF1-/-
A
0 3 6 12 24 36 48 0 3 6 12 24 36 48 TG (h, 300 nM)
GRP78
PERK
P-IREα
ATF6 (N)
XBP1s
XAF1
cl-PARP
Tubulin
F6
C
T47D-XAF1-/-
(Tet-XAF1)
0 3 6 12 24 TG + Tet
P-IREα
IRE1α
XBP1s
cl-PARP
XAF1
Tubulin

## Slide 9
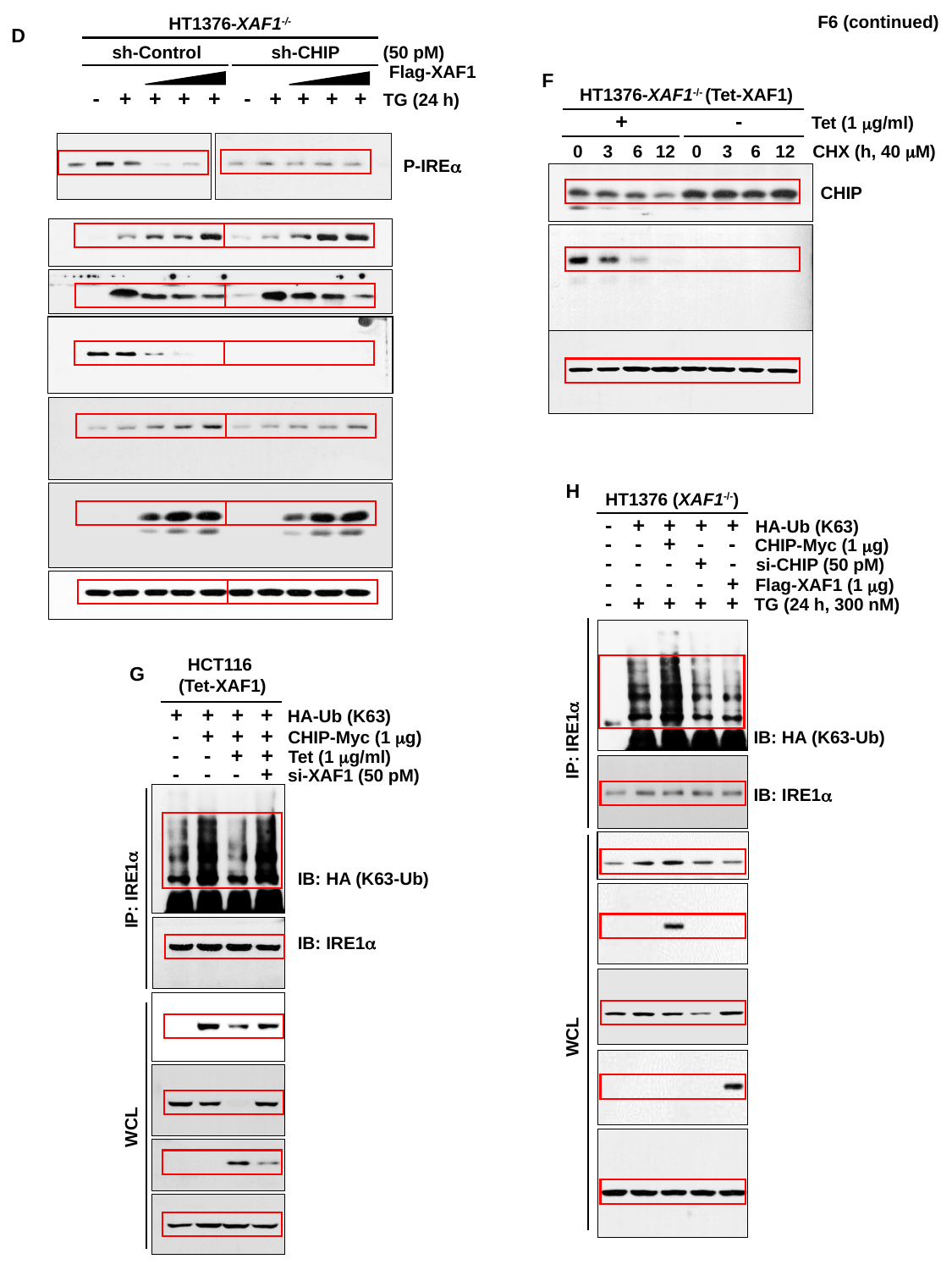

F6 (continued)
HT1376-XAF1-/-
D
Flag-XAF1
sh-Control sh-CHIP (50 pM)
- + + + + - + + + + TG (24 h)
P-IREα
IRE1α
GRP78
CHIP
cl-PARP
XAF1 (Flag)
Tubulin
F
HT1376-XAF1-/- (Tet-XAF1)
+ - Tet (1 μg/ml)
0 3 6 12 0 3 6 12 CHX (h, 40 μM)
CHIP
XAF1
Tubulin
H
HT1376 (XAF1-/-)
- + + + + HA-Ub (K63)
- - + - - CHIP-Myc (1 μg)
- - - + - si-CHIP (50 pM)
- - - - + Flag-XAF1 (1 μg)
- + + + + TG (24 h, 300 nM)
IB: HA (K63-Ub)
IB: IRE1α
P-IREα
CHIP (Myc)
CHIP
XAF1 (Flag)
Tubulin
IP: IRE1α
WCL
HCT116
(Tet-XAF1)
G
+ + + + HA-Ub (K63)
- + + + CHIP-Myc (1 μg)
- - + + Tet (1 μg/ml)
- - - + si-XAF1 (50 pM)
IB: HA (K63-Ub)
IB: IRE1α
CHIP (Myc)
CHIP
XAF1
Tubulin
IP: IRE1α
WCL

## Slide 10
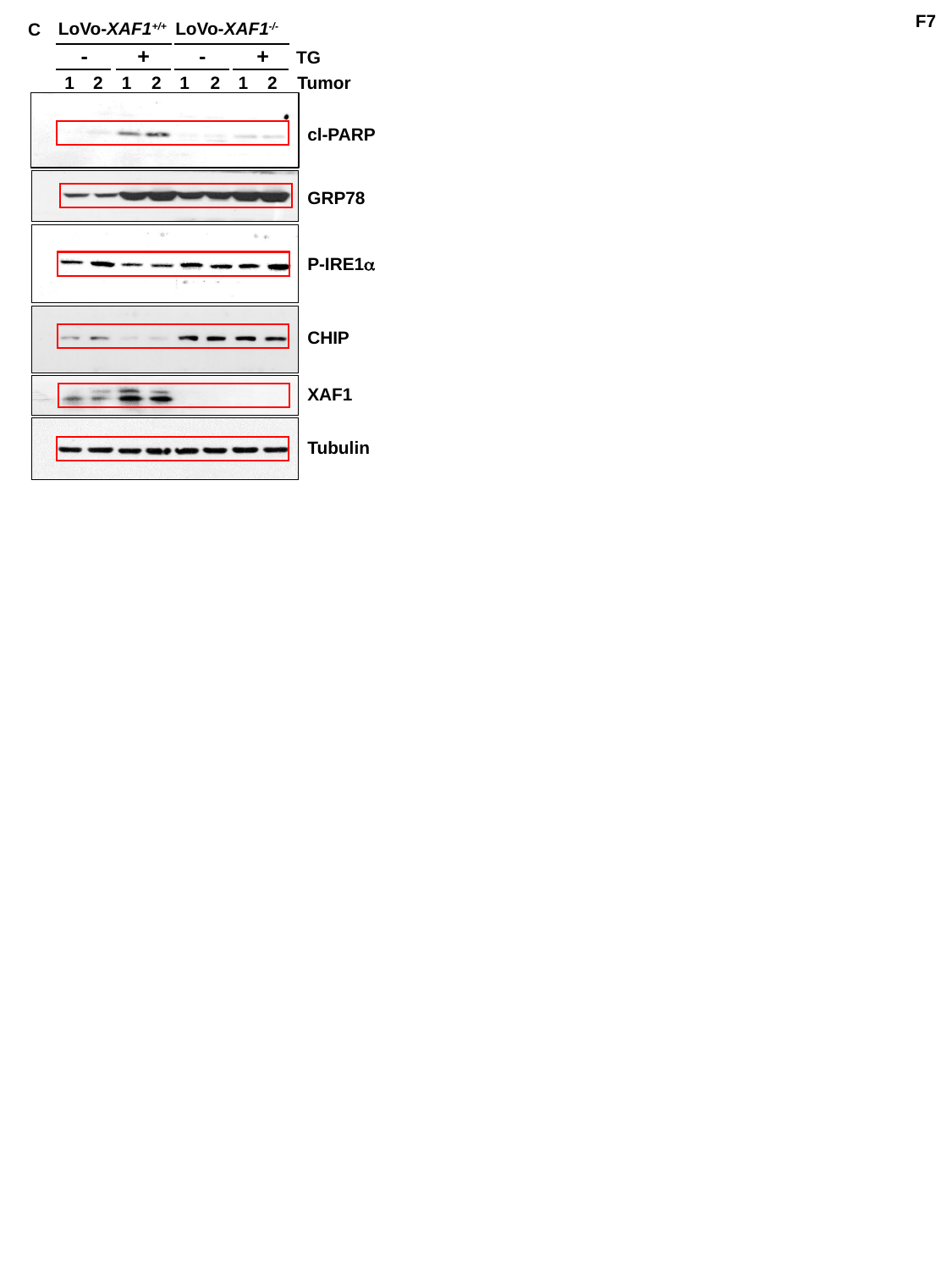

F7
LoVo-XAF1+/+ LoVo-XAF1-/-
C
- + - + TG
1 2 1 2 1 2 1 2 Tumor
cl-PARP
GRP78
P-IRE1α
CHIP
XAF1
Tubulin

## Slide 11
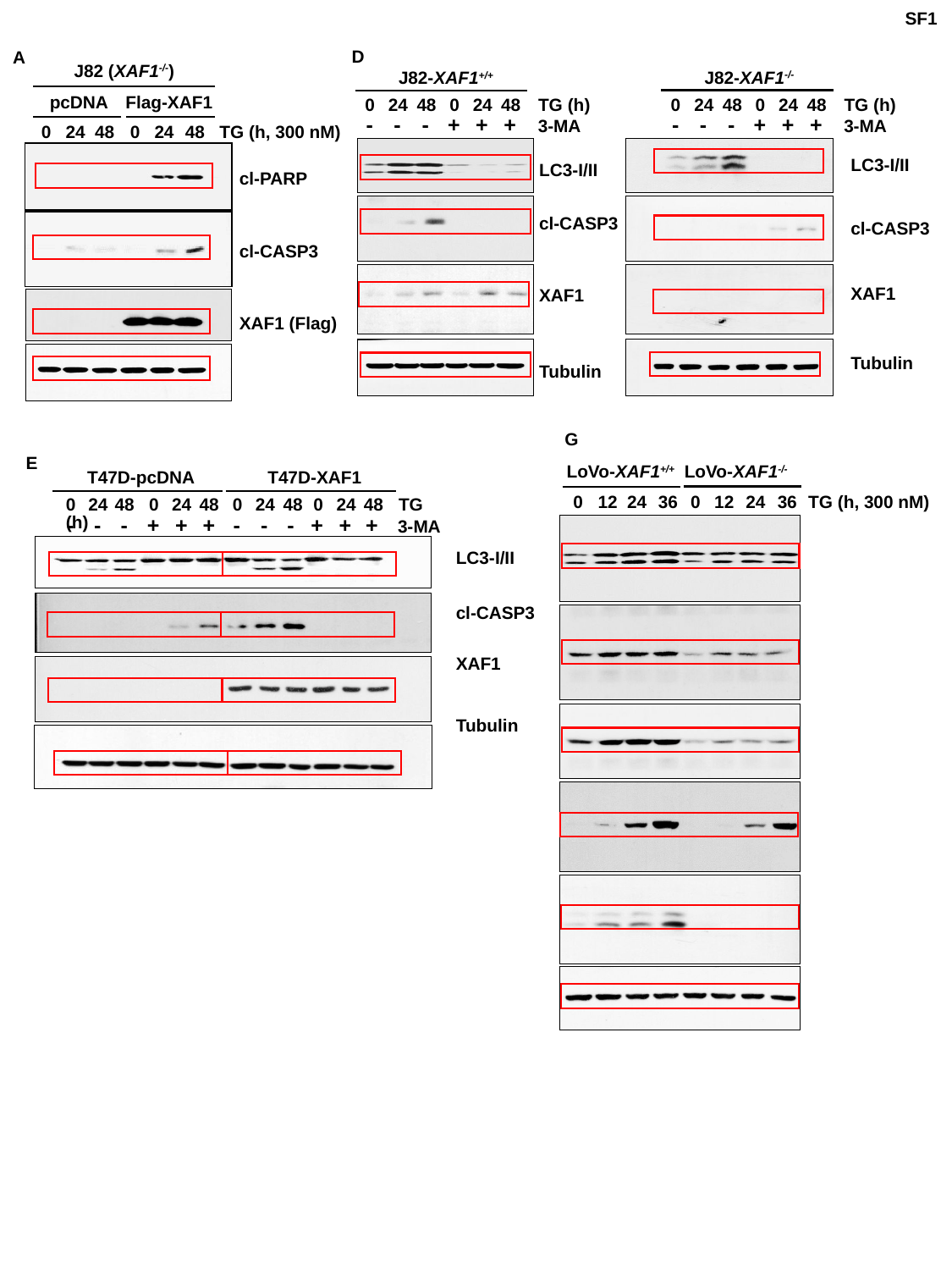

SF1
D
J82-XAF1+/+
0 24 48 0 24 48 TG (h)
- - - + + + 3-MA
J82-XAF1-/-
0 24 48 0 24 48 TG (h)
- - - + + + 3-MA
LC3-I/II
cl-CASP3
XAF1
Tubulin
LC3-I/II
cl-CASP3
XAF1
Tubulin
A
J82 (XAF1-/-)
 pcDNA Flag-XAF1
0 24 48 0 24 48 TG (h, 300 nM)
cl-PARP
cl-CASP3
XAF1 (Flag)
Tubulin
G
LoVo-XAF1+/+ LoVo-XAF1-/-
0 12 24 36 0 12 24 36 TG (h, 300 nM)
LC3-I/II
Beclin1
Atg5-Atg12
cl-PARP
XAF1
Tubulin
E
T47D-pcDNA T47D-XAF1
0 24 48 0 24 48 0 24 48 0 24 48 TG (h)
- - - + + + - - - + + + 3-MA
LC3-I/II
cl-CASP3
XAF1
Tubulin

## Slide 12
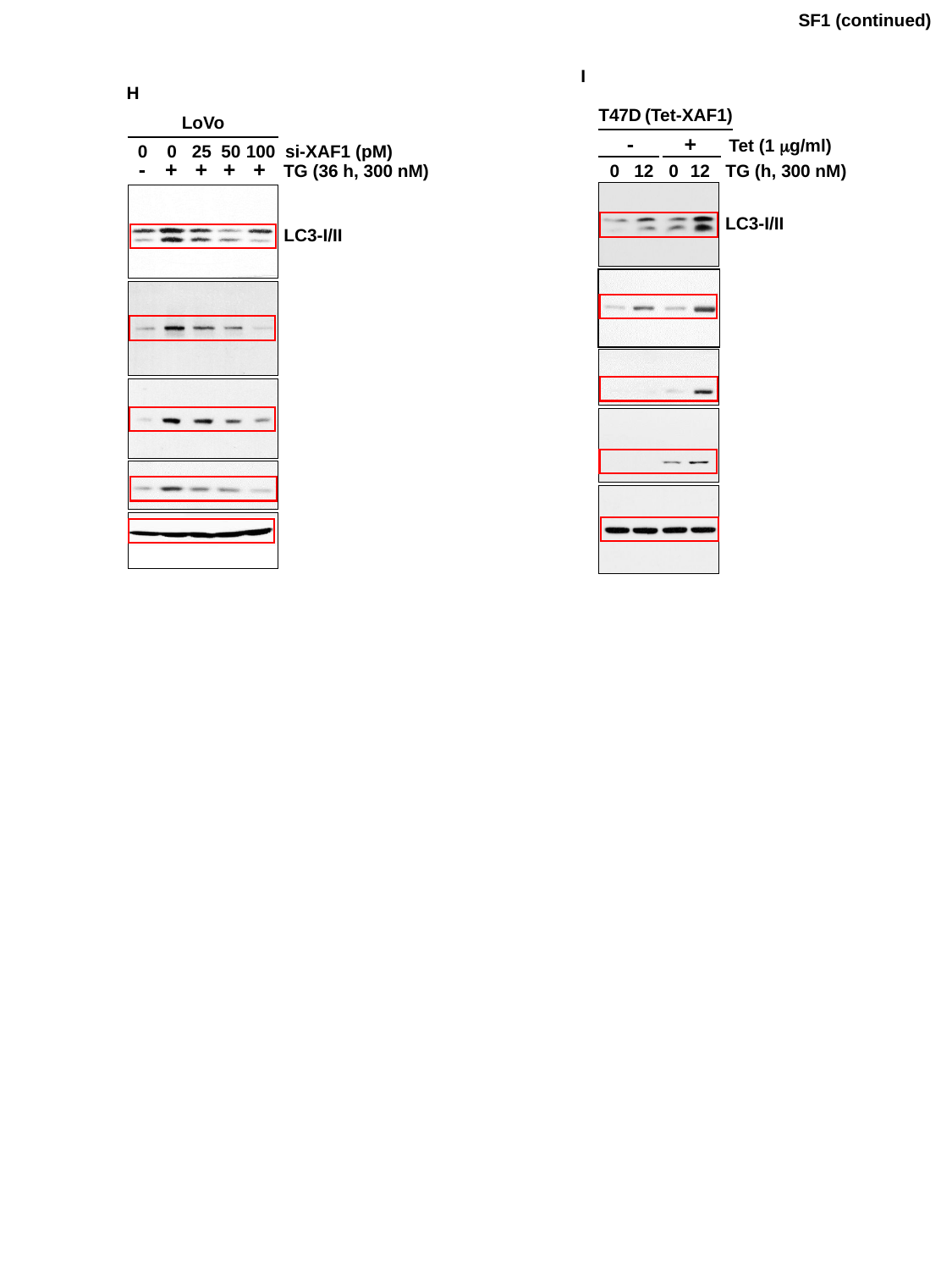

SF1 (continued)
I
T47D (Tet-XAF1)
- + Tet (1 μg/ml)
0 12 0 12 TG (h, 300 nM)
LC3-I/II
Beclin-1
cl-PARP
XAF1
Tubulin
H
LoVo
0 0 25 50 100 si-XAF1 (pM)
- + + + + TG (36 h, 300 nM)
LC3-I/II
Beclin-1
cl-PARP
XAF1
Tubulin

## Slide 13
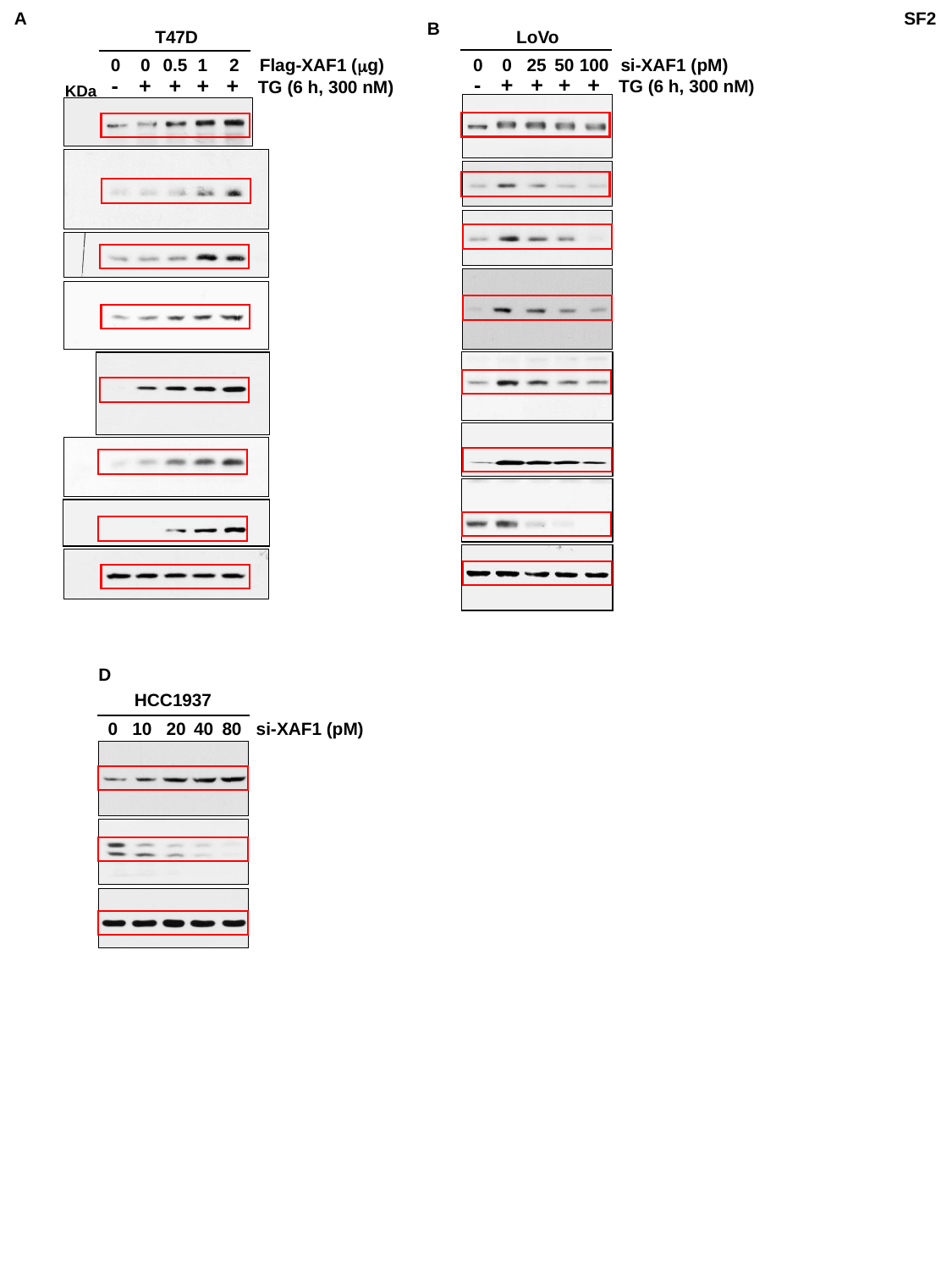

A
T47D
0 0 0.5 1 2 Flag-XAF1 (μg)
- + + + + TG (6 h, 300 nM)
KDa
PERK
P-IRE1α
ATF6 (N)
ATF4
P-eIF2α
CHOP
XAF1 (Flag)
Tubulin
SF2
B
LoVo
0 0 25 50 100 si-XAF1 (pM)
- + + + + TG (6 h, 300 nM)
PERK
P-IRE1α
ATF6 (N)
ATF4
P-eIF2α
CHOP
XAF1
Tubulin
D
HCC1937
0 10 20 40 80 si-XAF1 (pM)
GRP78
XAF1
Tubulin

## Slide 14
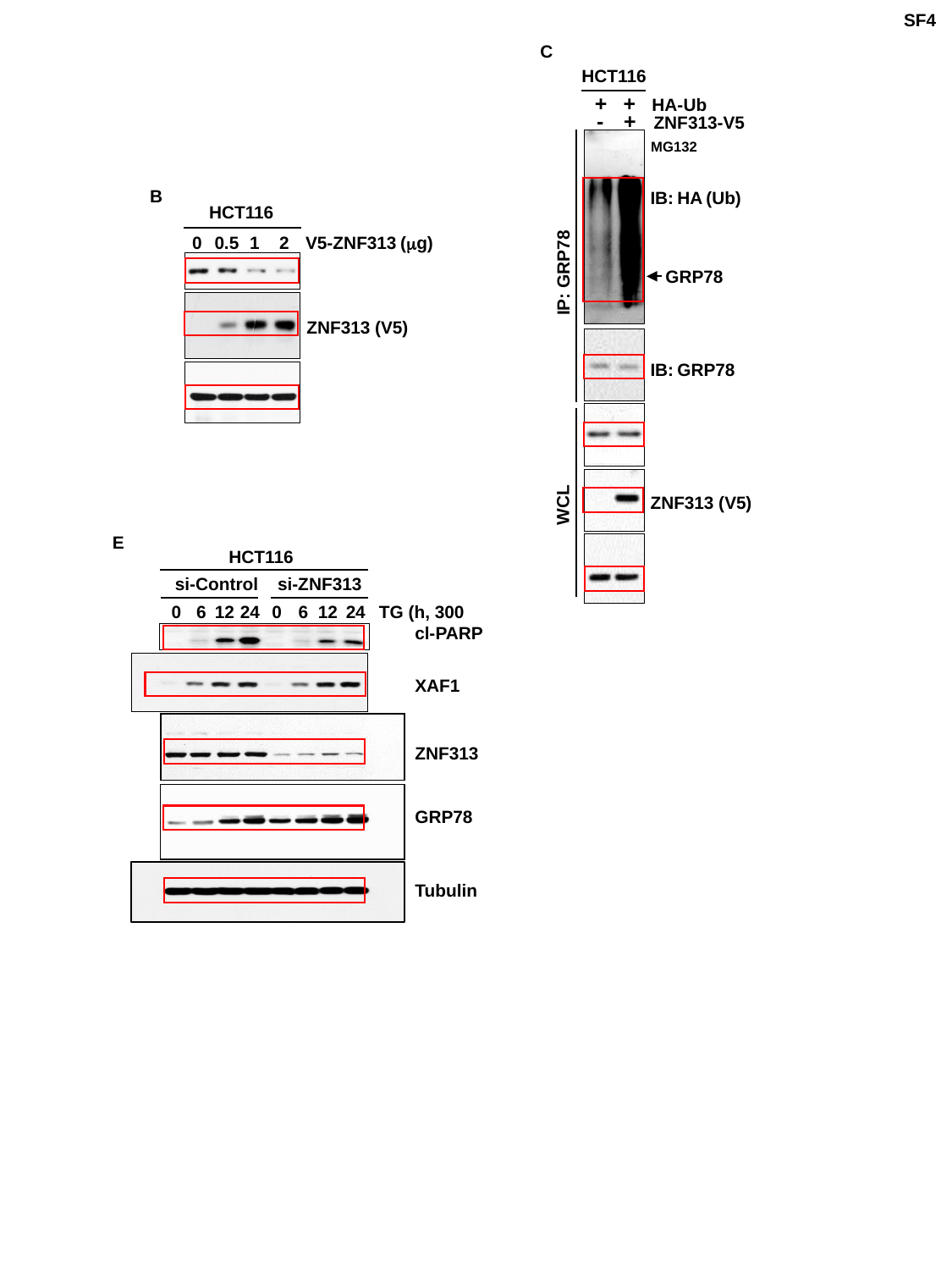

SF4
C
HCT116
+ + HA-Ub
- + ZNF313-V5
MG132
IB: HA (Ub)
IB: GRP78
 IP: GRP78
GRP78
GRP78
ZNF313 (V5)
Tubulin
WCL
B
HCT116
0 0.5 1 2 V5-ZNF313 (μg)
GRP78
ZNF313 (V5)
Tubulin
E
HCT116
si-Control si-ZNF313
0 6 12 24 0 6 12 24 TG (h, 300 nM)
cl-PARP
XAF1
ZNF313
GRP78
Tubulin

## Slide 15
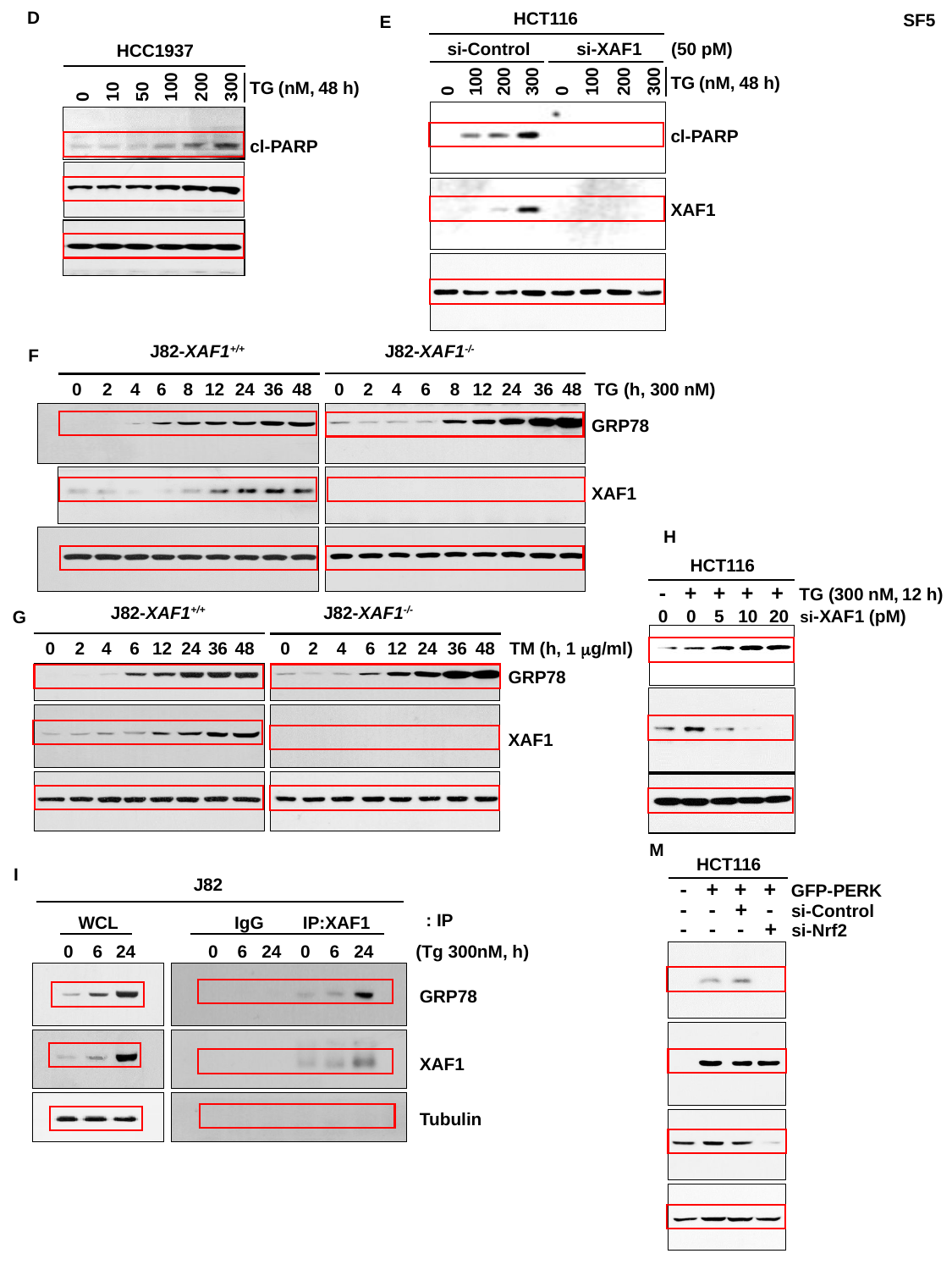

D
SF5
0
100
200
300
0
100
200
300
HCT116
E
si-Control si-XAF1 (50 pM)
TG (nM, 48 h)
cl-PARP
XAF1
Tubulin
0
10
50
100
200
300
HCC1937
TG (nM, 48 h)
cl-PARP
XAF1
Tubulin
J82-XAF1+/+ J82-XAF1-/-
0 2 4 6 8 12 24 36 48 0 2 4 6 8 12 24 36 48 TG (h, 300 nM)
GRP78
XAF1
Tubulin
F
H
HCT116
 - + + + + TG (300 nM, 12 h)
0 0 5 10 20 si-XAF1 (pM)
GRP78
XAF1
Tubulin
J82-XAF1+/+ J82-XAF1-/-
G
0 2 4 6 12 24 36 48 0 2 4 6 12 24 36 48 TM (h, 1 μg/ml)
GRP78
XAF1
Tubulin
M
HCT116
- + + + GFP-PERK
- - + - si-Control
- - - + si-Nrf2
XAF1
PERK (GFP)
Nrf2
Tubulin
I
J82
: IP
WCL IgG IP:XAF1
0 6 24 0 6 24 0 6 24 (Tg 300nM, h)
GRP78
XAF1
Tubulin

## Slide 16
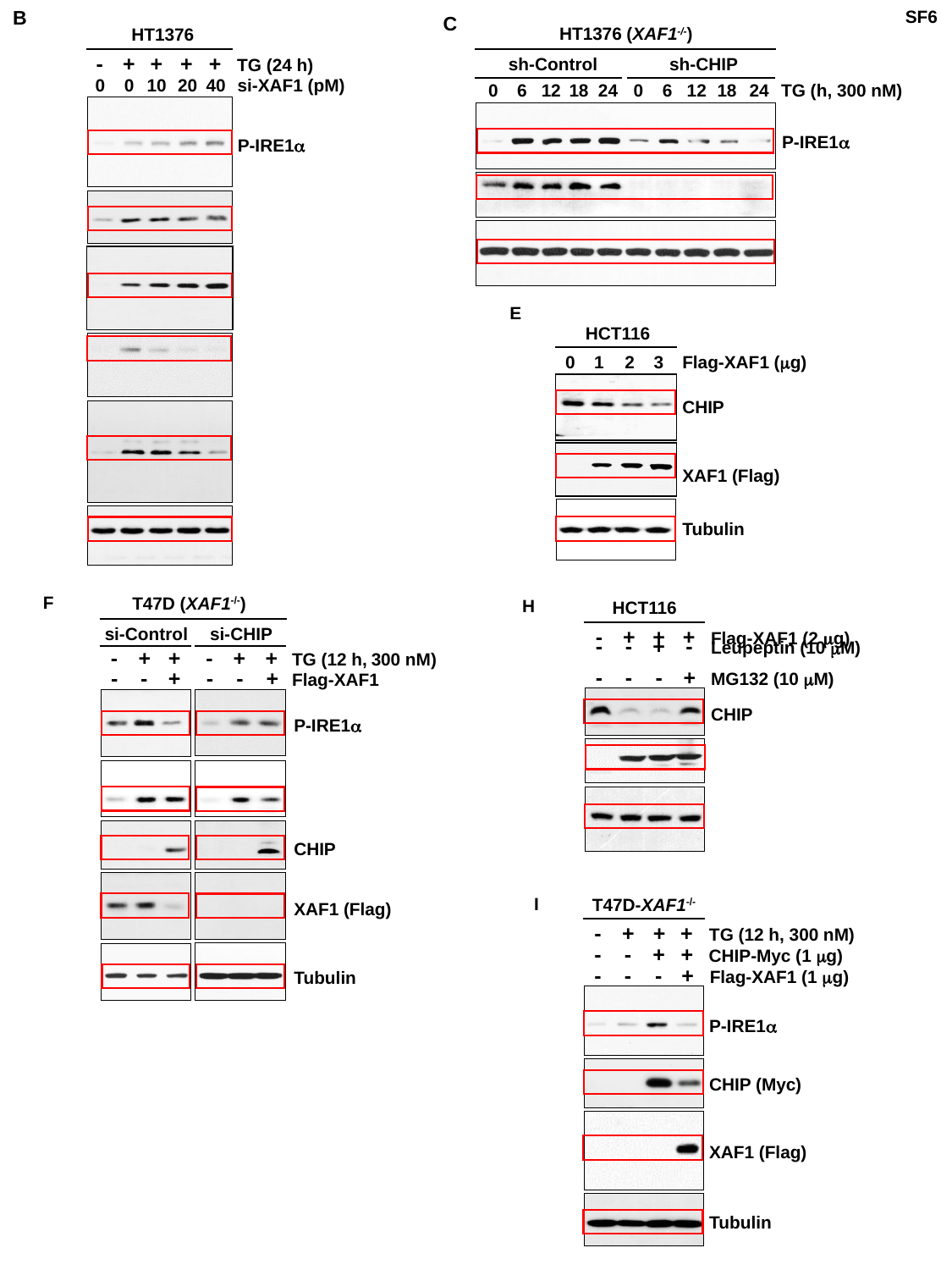

B
HT1376
 - + + + + TG (24 h)
0 0 10 20 40 si-XAF1 (pM)
P-IRE1α
IRE1α
XBP1s
cl-PARP
XAF1
Tubulin
SF6
C
HT1376 (XAF1-/-)
sh-Control sh-CHIP
0 6 12 18 24 0 6 12 18 24 TG (h, 300 nM)
P-IRE1α
CHIP
Tubulin
E
HCT116
0 1 2 3 Flag-XAF1 (μg)
CHIP
XAF1 (Flag)
Tubulin
F
T47D (XAF1-/-)
si-Control si-CHIP
- + + - + + TG (12 h, 300 nM)
- - + - - + Flag-XAF1
P-IRE1α
IRE1α
CHIP
XAF1 (Flag)
Tubulin
H
HCT116
- + + + Flag-XAF1 (2 μg)
- - + - Leupeptin (10 μM)
- - - + MG132 (10 μM)
CHIP
XAF1 (Flag)
Tubulin
I
T47D-XAF1-/-
- + + + TG (12 h, 300 nM)
- - + + CHIP-Myc (1 μg)
- - - + Flag-XAF1 (1 μg)
P-IRE1α
CHIP (Myc)
XAF1 (Flag)
Tubulin
